# Clinical practice guideline recommendations to improve the mental health of adult trauma patients: protocol for a systematic review

**DOI:** 10.1136/bmjopen-2023-079205

**Published:** 2024-03-25

**Authors:** Mélanie Bérubé, Nori Bradley, Meaghan O'Donnell, Henry Thomas Stelfox, Naisan Garraway, Helen-Maria Vasiliadis, Valerie Turcotte, Michel Perreault, Matthew Menear, Léonie Archambault, Juanita Haagsma, Hélène Provencher, Christine Genest, Marc-Aurèle Gagnon, Laurence Bourque, Alexandra Lapierre, Amal Khalfi, William Panenka

**Affiliations:** 1 Population Health and Optimal Practices Research Unit Research Unit (Trauma–Emergency–Critical Care Medicine), CHU de Québec-Université Laval Research Centre, Québec, Quebec, Canada; 2 Faculty of Nursing, Université Laval, Québec City, Quebec, Canada; 3 University of Alberta Hospital, Edmonton, Alberta, Canada; 4 Department of Psychiatry, The University of Melbourne Faculty of Medicine Dentistry and Health Sciences, Melbourne, Victoria, Australia; 5 Department of Critical Care Medicine, University of Calgary, Calgary, Alberta, Canada; 6 Vancouver General Hospital, Vancouver, British Columbia, Canada; 7 University of Sherbrooke, Longueil, Quebec, Canada; 8 Department of Social and Preventive Medicine, Université Laval, Québec City, Quebec, Canada; 9 Institut universitaire en santé mentale Douglas, Montréal, Quebec, Canada; 10 Department of Family Medicine and Emergency Medicine, Université Laval, Québec City, Quebec, Canada; 11 Faculty of Public Health, Erasmus MC, Rotterdam, The Netherlands; 12 Faculté des sciences infirmières, Université de Montréal, Montréal, Quebec, Canada; 13 Centre de recherche de l'Institut universitaire en santé mentale de Montréal, Montréal, Quebec, Canada; 14 Department of Psychiatry, The University of British Columbia, Vancouver, British Columbia, Canada

**Keywords:** TRAUMA MANAGEMENT, MENTAL HEALTH, Protocols & guidelines, Systematic Review

## Abstract

**Introduction:**

Mental disorders are common in adult patients with traumatic injuries. To limit the burden of poor psychological well-being in this population, recognised authorities have issued recommendations through clinical practice guidelines (CPGs). However, the uptake of evidence-based recommendations to improve the mental health of trauma patients has been low until recently. This may be explained by the complexity of optimising mental health practices and interpretating CGPs scope and quality. Our aim is to systematically review CPG mental health recommendations in the context of trauma care and appraise their quality.

**Methods and analysis:**

We will identify CPG through a search strategy applied to Medline, Embase, CINAHL, PsycINFO and Web of Science databases, as well as guidelines repositories and websites of trauma associations. We will target CPGs on adult and acute trauma populations including at least one recommendation on any prevention, screening, assessment, intervention, patient and family engagement, referral or follow-up procedure related to mental health endorsed by recognised organisations in high-income countries. No language limitations will be applied, and we will limit the search to the last 15 years. Pairs of reviewers will independently screen titles, abstracts, full texts, and carry out data extraction and quality assessment of CPGs using the Appraisal of Guidelines Research and Evaluation (AGREE) II. We will synthesise the evidence on recommendations for CPGs rated as moderate or high quality using a matrix based on the Grading of Recommendations Assessment, Development and Evaluation quality of evidence, strength of recommendation, health and social determinants and whether recommendations were made using a population-based approach.

**Ethics and dissemination:**

Ethics approval is not required, as we will conduct secondary analysis of published data. The results will be disseminated in a peer-reviewed journal, at international and national scientific meetings. Accessible summary will be distributed to interested parties through professional, healthcare quality and persons with lived experience associations.

**PROSPERO registration number:**

(ID454728).

**Strengths and limitations of this study**

- We will produce a meta-synthesis of clinical practice guidelines (CPGs) recommendations on mental health in injury care based on their methodological quality and level of evidence.
- Our review involves interested parties to maximise its relevance and reach.
- We will focus solely on CPGs for mental health from high-income countries.
- Our search strategy is not designed to identify CPGs that do not specifically focus on mental health in trauma populations.

## Introduction

Improvements in trauma care in high-income countries over the past 30 years have reduced mortality from as high as 50%–11%.^1 2^ As a result, more and more patients survive their traumatic injuries, but still face a significant burden of morbidity. Indeed, traumatic injuries are a leading cause of disability,^3 4^ and result in the highest number of productive years of life lost.^5 6^

The impact of traumatic injuries is not without consequences for patients’ mental health, not to mention the fact that many are already vulnerable on a psychosocial level. Before the injury, 20%–40% of trauma patients have mental health issues, including substance use disorders and mood disorders,^7 8^ which can impair recovery.^9^ After the injury, up to 45% of patients develop acute stress disorder during their hospital stay,^10^ and 10%–50% post-traumatic stress disorder in many high-income countries, with prevalence varying according to trauma mechanisms and recovery phases.^9 11–19^ Hence, patients who sustained traumatic injuries have a 40% higher risk of being hospitalised for a mental disorder when comparing the 5 years before and after the injury.^19^ Cumulative structural, social and health determinants have been shown to contribute significantly to mental disorders among trauma patients with racial/ethnic minorities, those with low levels of education and financial resources, males or patients identifying themselves as men and those living in rural areas being most vulnerable.^19 20^

The high prevalence of mental disorders in trauma patients is alarming considering that they have profound negative impacts on individual and social outcomes. Trauma patients suffering from mental disorders were shown to have twice as many complications and twice the length of hospital stays.^21^ Similarly, these patients are almost 10 times more likely to develop chronic pain and have physical limitations, and two to four times more likely not to return to work than patients without mental disorders,^22^ while having a poorer quality of life.^14^

Given the growing body of research evidence highlighting the burden of mental health issues following traumatic injury, recognised authorities^23–26^ have proposed clinical practice guidelines (CPGs) to reduce their prevalence. CPG recommendations apply to all healthcare professionals (HCPs) involved in the trauma care continuum, and cover mental health promotion/illness prevention, screening, brief and more comprehensive interventions, patient referral to subsequent specialty services and follow-up, and patient and family centered-care approach (ie, collaborative approach, share-decision making^27^). HCPs’ adherence rates to CPGs just prior to the publication of key CPGs were issued in late 2022, such as the American College of Surgeons-Committee on Trauma on Screening and Intervention for Substance Use in the Acute Trauma Patients,^23^ were are reported to be less than 30%.^28 29^ While uptake rates may have risen since then, the fact remains that improving mental health practices in an area where HCPs have more expertise in physical health^19 30^ can be complex and put pressure on increasingly limited resources of healthcare systems. It is therefore crucial to summarise the recommendations on the prevention and treatment of mental disorders in the context of trauma, and identified those with the greatest support for implementation, to enable organisations in different countries to target the priorities on which to focus. Hence, we aim to systematically review CPG recommendations for mental health in the context of injury care, and to appraise their quality.

## Methods

In line with the knowledge synthesis phase of the Knowledge to Action framework,^31^ we will conduct a knowledge synthesis on CPGs. This protocol was developed according to Cochrane recommendations for systematic reviews (SR)^32^ and methodological guidelines for SR on CPGs^33^ and is reported according to the Preferred Reporting Items for Systematic review and Meta-Analysis Protocol statement.^34^

### Patient and public involvement

To maximise contributions, we set up an advisory committee including representatives of associations that play a role in setting standards for trauma and mental healthcare (Trauma Association of Canada, the Mental Health Commission of Canada, and the *Institut national d’excellence en santé et en services sociaux*) and in supporting persons with lived experience (Brain Injury Canada, Mothers Against Drunk Driving—Canada, *Moelle épinière et motricité Québec and Connexion TCC-Québec*). The advisory committee also includes HCPs from various disciplines involved with trauma patients (specialised physicians/surgeons, nurses, rehabilitation professionals, psychologists and social workers), persons with lived experience and decision makers. The committee was involved in the development of the protocol and will oversee the progress of the review and take part in disseminating the results.

### Eligibility

We will target CPGs on adult (≥18 years old) and in-patient acute trauma populations (P) including at least one recommendation (R) on any prevention, screening, assessment, intervention, patient and family engagement, referral or follow-up procedure related to mental health (I) with any or no comparator (C) endorsed by recognised organisations in high-income countries within the last 15 years (September 2008 to a maximum of 6 months prior to submission) (A). No language restriction will be applied. We will use an open-access online translator (https://www.deepl.com/translator) for studies not written in English of French.^35^ CGPs are defined as ‘statements that include recommendations intended to optimise patient care that are informed by a review of evidence and an assessment of benefits and harms of alternative care options’.^36^ We will include CPGs focusing on the categories of mental disorders defined in the Diagnostic and Statistical Manual of Mental Disorders fifth edition-text revision (DSM-5-TR)^37^ that most often affect trauma patients: trauma-and-stressor-related disorders, anxiety disorders, substance-related and addictive disorders, suicidal behaviour disorder and self-injury.^7 8 19^ High-income countries, based on World Bank definitions,^38^ will be targeted to ensure that recommendations are compatible with the practices of accredited trauma centres,^39^ while the time limit aims to focus on recent recommendations.

### Search strategy

Based on the recommendations of the Peer Review of Electronic Search Strategies,^40^ we will establish a systematic search strategy in collaboration with an information specialist, which will be independently reviewed by a second information specialist. We will use a combination of controlled terms (eg, MeSH for MEDLINE and Emtree for Embase) and free vocabulary according to the following themes: wounds and injuries, trauma, mental health, mental disorders and guidelines. We will apply the pilot search strategy in Medline (Ovid) and Embase (Ovid) databases. Relevant words found in the title, abstract and text of retrieved studies will be collected. We will then add these words and indexing terms to the search strategy, which will be executed in the Medline (Ovid), Embase (Ovid), CINAHL (EBSCO), PsycINFO (Ovid) and Web of Science (Clarivate) databases. Relevant GPGs will also be searched in CPGs repositories and trauma association websites, a list of which was drawn up with members of the research team who have in-depth knowledge of the innerworkings in this field. Using a preliminary search strategy (from January 2018 to 8 August 2023; Tables 1 and 2), we have identified 3882 citations, including five sentinel CPGs^24–26 41 42^ identified a priori, indicating good sensitivity and specificity.

**Table 1:** Medline (Ovid) (8 August 2023)

Concepts	Search strategy keywords	Research and no of results
Trauma/recovery (controlled vocabulary) (free text)	exp ‘Wounds and Injuries’/ or exp Brain Hemorrhage, Traumatic/ or Brain Injuries/ or Coma, Post-Head Injury/ or Craniocerebral Trauma/ or Diffuse Axonal Injury/ or exp Fractures, Bone/ or Head Injuries, Closed/ or Head Injuries, Penetrating/ or exp Intracranial Hemorrhage, Traumatic/ or exp Skull Fractures/	#1 1 007 620
	Fractur* OR Injur* OR TBI OR trauma*	#2 1 430 550
	1 OR 2	#3 1 894 323
Mental health (controlled vocabulary) (free text)	exp Mental Disorders/ or exp Mental Health/ or exp Substance-Related Disorders/	#4 1 501 202
	‘mental health’ OR ‘mental disorder*’ OR ‘mental illness’ OR ‘post-traumatic stress disorder’ OR ‘post traumatic stress disorder’ OR ‘PTSD’ OR ‘Acute stress disorder*’ or ‘acute stress reaction*’ or ‘adjustment disorder*’ or ‘suicidal behavior*’ or ‘self-injur*’ or ‘self injur*’ or ‘self-harm’ or ‘self harm’ or ‘depressive disorder*’ or ‘depression’ or ‘mood disorder*’ or ‘anxiety disorder*’ or ‘trauma and stressor-related disorder*’ or ‘trauma and stressor related disorder*’ or ‘Mental distress’ or ‘psychological distress’ OR ((drug* OR substance OR alcohol OR marijuana OR cannabis OR narcotic* OR opiate* OR opioid* OR opium) Adj4 (abus* OR ‘use’ OR user OR usage OR misus* OR usin* OR utilis* OR depend* OR addict* OR illegal* OR illicit* OR habit* OR withdraw* OR behavi* OR abstinence* OR abstain* OR intoxica* OR addict* OR disorder*)) OR ‘drug rehabilitation’ OR ‘non‐prescription drugs’ OR ‘non‐prescription drug’	#5 1 069 467
	4 OR 5	#6 2 121 781
Guideline (controlled vocabulary) (free text)	exp Practice Guidelines as Topic/ or exp Practice Guideline/ or exp Guidelines as Topic/ or exp Guideline/	#7 208 566
	Guide OR Guideline*	#8 676 237
	7 OR 8	#9 784 520
Total	3 AND 6 AND 9 Limit to 2008–2023	3882

PTSD, post-traumatic stress disorder.

**Table 2:** Preliminary list of clinical practice guidelines repositories and trauma professional associations

1-Agency for Healthcare Research and Quality	32-Guideline Central
2-Accreditation Canada	33-Guidelines International Network
3-Australasian Association for Quality in Healthcare	34-Headway—the brain injury association
4-American Academy of Orthopaedic Surgeons	35-International Association for Trauma Surgery and Intensive Care
5-American Association for the Surgery on Trauma	36-International Society of Orthopaedic and Traumatology
6-American Association of Neurological Surgeons	37-Italian Society of Orthopaedics and Traumatology
7-American Board of Orthopaedic Surgery	38-National Association for Healthcare Quality
8-American College of Surgeons	39-National Guidelines Clearinghouse
9-American Orthopaedic Association	40-National Institute of Health and Care Excellence
10-American Spinal Injury Association	41-National Quality Forum
11-American Trauma Society	42-Norwegian Neurosurgical Association
12-Australian Society of Orthopeadic Surgeons	43-Ontario Neurotrauma Foundation
13-Australian and New Zealand Society	44-Orthopaedic Trauma Association
14-Belgian Society for Orthopaedics and Traumatology	45-Scandinavian Neurotrauma Committee
15-Brain Injury Association of America	46-Scottish Intercollegiate Guidelines Network
16-Brain Trauma Foundation	47-Société Française de Chirurgie Orthopédique et Traumatologique
17-British Orthopaedic Association	48-Society of Critical Care Medicine Trauma Network
18-British Trauma Society	49-Société Royale Belge de Chirurgie Orthopédique et de Traumatologie
19-Canada’s Drug and Health Technology Agency	50-Spanish Society of Orthopaedic Surgery and Traumatology
20-Centres for Disease Control and Prevention	51-Society of Trauma Nurses
21-Congress of Neurological Surgeons	52-Spinal Cord Injury Research Evidence
22-Consortium of Spinal Cord Medicine	53-Substance Abuse and Mental Health Services Administrations
23-Danish Neurosurgical Society	54-Swedish Neurosurgical Societ
24-Dutch Institute for Healthcare Improvement	55-Trauma Association of Canada
25-Eastern Association for the Surgery of Trauma	56-Trauma Audit Research Network
26-European Association of Neurosurgical Societies	57-Trauma.org
27-European Federations of National Associations of Orthopaedic and Traumatology	58-US Department of Health and Human Services
28-European Society for Trauma and Emergency Surgery	59-US Department of Veterans Affairs
29-Finnish Association of Neuroscience Nurses	60-Western Trauma Association
30-Finnish Neurosurgical Society	61-World Health Organisation
31-Greek Society of Orthopaedic and Traumatological Surgery	

### CPGs selection and data extraction

We will manage citations using EndNote (V.X9.3.3, New York City: Thomson Reuters, 2018). Pairs of reviewers will independently screen titles, abstracts and full-texts, and carry out data extraction. Once the final set of included CPGs has been obtained, all associated methodological supplements will be retrieved before data extraction or quality assessment begins.^33^ Data from included CPGs will be extracted with an extraction form, which will be pilot-tested on five items to ensure feasibility and completeness.^32^ For each recommendation within CPGs, we will extract information on the characteristics of included CPGs: title and year, country, organisation, target users, population including determinants contributing to disparities in mental healthcare^43 44^ (eg, type of trauma, gender, age group, ethnocultural group, geographical location and socioeconomic status), areas of focus, intervention, quality of evidence and strength of recommendations. Since interventions that use a population-based approach, that is, stepped and collaborative care tailored to patients’ risks and needs, have been shown to be more effective than standardised clinical interventions in improving the mental health of trauma patients,^45^ we will also extract data on whether recommendations provide details on these aspects. If important information is missing or unclear, we will request it by sending up to three emails to the first, second and last authors.

### Quality

Pairs of reviewers with content expertise will independently assess CPGs quality based on the six domains of the AGREE II tool:^46^ (1) scope and purpose (overall aim of the guideline; specific health questions and target population), (2) interested parties involvement (developed by the appropriate stakeholders; consistent with the views of its intended users), (3) rigorously developed (process used to gather and synthesise the evidence; methods to formulate and update the recommendations), (4) clarity and presentation (language; structure and format), (5) applicability (barriers and facilitators to implementation; strategies to improve uptake such as integration into a national trauma performance verification process, tools for use at an organisational level, billing guidance for mental health practices, links to other relevant guidelines and tools and demand on healthcare resources) and (6) editorial independence (competing interests reported and addressed). In accordance with guidelines on the SR of CPGs^33^ and based on previous SRs of CPGs,^47–49^ each domain with a score≥60% will be considered effectively addressed. CPGs will be considered *high quality* if they score≥60% in at least three of the six AGREE II domains, including domain 3 (rigour of development). If three domains or more score ≥60%, and domain 3 score<60%, the CPG will be considered *moderate quality*. CPGs scoring<60% in two domains or more and scoring<50% in domain 3 will be considered *low quality*.

### Data synthesis

CPG recommendations will be classified into the following key areas, as determined by the advisory committee: (1) mental health promotion/illness prevention, (2) screening for mental disorders, (3) assessment, (4) interventions, (5) referral for patients experiencing mental disorders for follow-up and support services and (6) patient and family centered-care approach. We will synthesise the evidence on recommendations for CPGs rated as moderate or high quality (AGREE II)^46^ using a recommendation matrix based on the Grading of Recommendations Assessment, Development and Evaluation (GRADE) quality of evidence (high, moderate, low and very low),^50^ recommendation strength (low and high),^47–49^ health and social determinants (ie, care equity), and whether or not recommendations were formulated using a population-based approach.^45^ Matrix data will be extracted independently by pairs or reviewers for each recommendation. We will group the same or very similar recommendations published by more than one CPG. For CPGs that used a rating system other than GRADE, we will match reported categories to GRADE categories.

For selection, extraction, quality assessment and meta-synthesis, all reviewers will systematically and iteratively pilot test the process on a random selection of articles (200 citations for selection and five CPGs for extraction and quality evaluation and meta-synthesis in each iteration) until acceptable agreement is achieved (kappa>0.8).^32^ Any discrepancies during the review process will be resolved by first consulting a senior member of the research team (WP), and then by reaching a consensus among the members of the advisory committee, if necessary.

### Limitation of study

For feasibility reasons, our search strategy was not developed to systematically identify CPGs that do not specifically target mental disorders in trauma populations. Therefore, we may miss recommendations on mental health if they are included in CPGs that target trauma populations in general if no keywords relating to mental health and injury are present in the title or abstract. However, these recommendations are likely to be identified by consulting CPGs repositories and trauma association websites (table 2), which were recognised as the best sources to find relevant guidelines.^51^

## Potential impact

Recent data on mental health issues in trauma populations are now prompting recognised authorities^23–26^ to prioritise this area to further improve the outcomes in injured patients. However, to date, there has been no synthesis of recommendations for improving mental health practices in the trauma field. This SR of CPGs will allow to identify recommendations with the greatest support for implementation, thus taking into account the staff shortage and time constraints omnipresent in healthcare systems. Results may be used by HCPs, trauma programme leaders, hospital administrators and policymakers to inform local quality improvement initiatives, identify system-wide problems and plan future actions to improve mental health practices.

## Ethics and dissemination

Research ethics approval is not required, as we will conduct secondary analysis of published data. The results of our study will be disseminated in a peer-reviewed journal, at international and national scientific meetings, and an accessible summary will be distributed to stakeholders through professional, healthcare quality and persons with lived experience associations.

## Supplementary Material

Reviewer comments

Author's manuscript

## References

[1] Moore L , Evans D , Hameed SM , et al . Mortality in Canadian trauma systems: a multicenter cohort study. Ann Surg 2017;265:212–7. 10.1097/SLA.0000000000001614 28009748

[2] Truchon C , Moore L , Belcaid A , et al . Shaping quality through vision, structure, and monitoring of performance and quality indicators: impact story from the Quebec trauma network. Int J Technol Assess Health Care 2017;33:415–9. 10.1017/S0266462317000198 28578738

[3] World Health Organization . WHO reveals leading causes of death and disability worldwide: 2000-2019. 2020. Available: https://www.who.int/news/item/09-12-2020-who-reveals-leading-causes-of-death-and-disability-worldwide-2000-2019

[4] Vos T , Lim SS , Abbafati C , et al . Diseases and injuries collaborators. global burden of 369 diseases and injuries in 204 countries and territories, 1990–2019: a systematic analysis for the global burden of disease study 2019. The Lancet 2020;396:1204–22. 10.1016/S0140-6736(20)30925-9 PMC7567026 33069326

[5] Parachute . Cost of injury in Canada. 2022. Available: https://parachute.ca/en/professional-resource/cost-of-injury-in-canada/

[6] Centers for Disease Control and Prevention . Injury prevention & control: cost of injury data. 2021. Available: https://www.cdc.gov/injury/wisqars/cost/

[7] Clous EA , Beerthuizen KC , Ponsen KJ , et al . Trauma and psychiatric disorders: a systematic review. J Trauma Acute Care Surg 2017;82:794–801. 10.1097/TA.0000000000001371 28129262

[8] Weinberg DS , Narayanan AS , Boden KA , et al . Psychiatric illness is common among patients with orthopaedic polytrauma and is linked with poor outcomes. J Bone Joint Surg Am 2016;98:341–8. 10.2106/JBJS.15.00751 26935455

[9] Kruithof N , Polinder S , de Munter L , et al . Health status and psychological outcomes after trauma: a prospective multicenter cohort study. PLoS One 2020;15:e0231649. 10.1371/journal.pone.0231649 32315373 PMC7173764

[10] Wiseman T , Foster K , Curtis K . Mental health following traumatic physical injury: an integrative literature review. Injury 2013;44:1383–90. 10.1016/j.injury.2012.02.015 22409991

[11] Lin W , Gong L , Xia M , et al . Prevalence of Posttraumatic stress disorder among road traffic accident survivors: A PRISMA-compliant meta-analysis. Medicine (Baltimore) 2018;97:e9693. 10.1097/MD.0000000000009693 29505023 PMC5779792

[12] Roden-Foreman K , Solis J , Jones A , et al . Prospective evaluation of posttraumatic stress disorder and depression in orthopaedic injury patients with and without concomitant traumatic brain injury. J Orthop Trauma 2017;31:e275–80. 10.1097/BOT.0000000000000884 28832389

[13] Joseph NM , Benedick A , Flanagan CD , et al . Risk factors for posttraumatic stress disorder in acute trauma patients. J Orthop Trauma 2021;35:e209–15. 10.1097/BOT.0000000000001990 33724967

[14] van der Vlegel M , Polinder S , Toet H , et al . Anxiety, depression and post-traumatic stress symptoms among injury patients and the association with outcome after injury. Eur J Psychotraumatol 2022;13:2023422. 10.1080/20008198.2021.2023422 35111285 PMC8803064

[15] Warren AM , Jones AL , Bennett M , et al . Prospective evaluation of Posttraumatic stress disorder in injured patients with and without Orthopaedic injury. J Orthop Trauma 2016;30:e305–11. 10.1097/BOT.0000000000000623 27253481

[16] Bryant RA , O’Donnell ML , Creamer M , et al . The psychiatric sequelae of traumatic injury. Am J Psychiatry 2010;167:312–20. 10.1176/appi.ajp.2009.09050617 20048022

[17] Zatzick DF , Rivara FP , Nathens AB , et al . A nationwide US study of post-traumatic stress after hospitalization for physical injury. Psychol Med 2007;37:1469–80. 10.1017/S0033291707000943 17559704

[18] Herrera-Escobar JP , Al Rafai SS , Seshadri AJ , et al . A multicenter study of post-traumatic stress disorder after injury: mechanism matters more than injury severity. Surgery 2018;164:1246–50. 10.1016/j.surg.2018.07.017 30170820

[19] Evans CCD , DeWit Y , Seitz D , et al . Mental health outcomes after major trauma in Ontario: a population-based analysis. CMAJ 2018;190:E1319–27. 10.1503/cmaj.180368 30420387 PMC6232008

[20] Mollayeva T , Mollayeva S , Pacheco N , et al . Systematic review of sex and gender effects in traumatic brain injury: equity in clinical and functional outcomes. Front Neurol 2021;12:678971. 10.3389/fneur.2021.678971 34566834 PMC8461184

[21] Sharwood LN , Wiseman T , Tseris E , et al . Pre-existing mental disorder, clinical profile, inpatient services and costs in people hospitalised following traumatic spinal injury: a whole population record linkage study. Inj Prev 2020. 10.1136/injuryprev-2019-043567 32414771

[22] Herrera-Escobar JP , Seshadri AJ , Stanek E , et al . Mental health burden after injury: it’s about more than just posttraumatic stress disorder. Ann Surg 2021;274:e1162–9. 10.1097/SLA.0000000000003780 32511129

[23] Committee on Trauma-American College of Surgeons . Best practices guidelines: screening and interventions for mental health disorders and subctance use in the acute trauma patients, . 2022 Available: https://www.facs.org/quality-programs/trauma/quality/best-practices-guidelines/

[24] Substance Abuse and Mental Health Services Administration . Substance use disorder treatment for people with physical and cognitive disabilities advisory. 2019. Available: https://store.samhsa.gov/sites/default/files/d7/priv/pep19-02-00002_508_022620.pdf 22514835

[25] Bombardier CH , Azuero CB , Fann JR , et al . Management of mental health disorders, substance use disorders, and suicide in adults with spinal cord injury: clinical practice guideline for Healthcare providers. Top Spinal Cord Inj Rehabil 2021;27:152–224. 10.46292/sci2702-152 34108836 PMC8152173

[26] Major Extremity trauma and Rehabilitation Consortium and the American Academy of Orthopaedic Surgeons . Evaluation of psychosocial factors influencing recovery from adult orthopeadic trauma, . 2019 Available: https://www.aaos.org/globalassets/quality-and-practice-resources/dod/prf-cpg-final-draft-12-3-19.pdf 10.5435/JAAOS-D-21-00777 34714783

[27] World Health Organization . WHO global strategy on people-centred and integrated health services. 2015. Available: https://interprofessional.global/wp-content/uploads/2019/11/WHO-2015-Global-strategy-on-integrated-people-centred-health-services-2016-2026.pdf

[28] Lamontagne M-E , Gargaro J , Marier-Deschênes P , et al . A survey of perceived implementation gaps for a clinical practice guideline for the rehabilitation of adults with moderate to severe traumatic brain injury. J Head Trauma Rehabil 2018;33:306–16. 10.1097/HTR.0000000000000430 30188460

[29] Bulger EM , Johnson P , Parker L , et al . Nationwide survey of trauma center screening and intervention practices for posttraumatic stress disorder, firearm violence. J Am Coll Surg 2022;234:274–87. 10.1097/XCS.0000000000000064 35213489 PMC10234338

[30] Sinkler MA , Furdock RJ , Vallier HA . Treating trauma more effectively: a review of psychosocial programming. Injury 2022;53:1756–64. 10.1016/j.injury.2022.04.022 35491278

[31] Graham ID , Logan J , Harrison MB , et al . Lost in knowledge translation: time for a map J Contin Educ Health Prof 2006;26:13–24. 10.1002/chp.47 16557505

[32] Higgins JT , Chandler J , Cumpston M , et al . Cochrane Handbook for systematic reviews of interventions version 6.3. 2022. Available: www.training.cochrane.org/handbook

[33] Johnston A , Kelly SE , Hsieh S-C , et al . Systematic reviews of clinical practice guidelines: a methodological guide. J Clin Epidemiol 2019;108:64–76. 10.1016/j.jclinepi.2018.11.030 30529647

[34] Moher D , Shamseer L , Clarke M , et al . Preferred reporting items for systematic review and meta-analysis protocols (PRISMA-P) 2015 statement. Syst Rev 2015;4:1. 10.1186/2046-4053-4-1 25554246 PMC4320440

[35] Takakusagi Y , Oike T , Shirai K , et al . Validation of the reliability of machine translation for a medical article from Japanese to English using Deepl translator. Cureus 2021;13:e17778. 10.7759/cureus.17778

[36] Institute of Medicine (US) Committee on Standards for Developing Trustworthy Clinical Practice Guidelines . Clinical practice guidelines we can trust. Washington (DC): National Academies Press (US), 2011.

[37] American Psychiatric Association . Diagnostic and Statistical Manual of Mental Disorders. Washington (DC), US, 2022. 10.1176/appi.books.9780890425787

[38] Blogs WB . New World Bank country classifications by income level: 2022-2023. 2023. Available: https://blogs.worldbank.org/opendata/new-world-bank-country-classifications-income-level-2022-2023

[39] Committee on Trauma-American College of Surgeons . Resources for optimal care of the injured patient. 2022. Available: https://www.facs.org/quality-programs/trauma/quality/verification-review-and-consultation-program/standards/

[40] McGowan J , Sampson M , Salzwedel DM , et al . PRESS peer review of electronic search strategies: 2015 guideline statement. J Clin Epidemiol 2016;75:40–6. 10.1016/j.jclinepi.2016.01.021 27005575

[41] Metha SOS , Blackport D , Teasell R . Mental health after a spinal cord injury. 2022. Available: https://scireproject.com/evidence/mental-health/introduction/

[42] Kodadek LM , Freeman JJ , Tiwary D , et al . Alcohol-related trauma reinjury prevention with hospital-based screening in adult populations: an Eastern Association for the surgery of trauma evidence-based systematic review. J Trauma Acute Care Surg 2020;88:106–12. 10.1097/TA.0000000000002501 31490336

[43] Haider AH , Weygandt PL , Bentley JM , et al . Disparities in trauma care and outcomes in the. United States: A. systematic review and meta-analysis. J Trauma Acute Care Surg 2013;74:1195–205. 10.1097/TA.0b013e31828c331d 23609267 PMC3641534

[44] Englum BR , Villegas C , Bolorunduro O , et al . Racial, ethnic, and insurance status disparities in use of posthospitalization care after trauma. J Am Coll Surg 2011;213:699–708. 10.1016/j.jamcollsurg.2011.08.017 21958511 PMC3684147

[45] Giummarra MJ , Lennox A , Dali G , et al . Early psychological interventions for posttraumatic stress, depression and anxiety after traumatic injury: a systematic review and meta-analysis. Clin Psychol Rev 2018;62:11–36. 10.1016/j.cpr.2018.05.001 29754102

[46] Brouwers MC , Kho ME , Browman GP , et al . AGREE II: advancing guideline development, reporting and evaluation in health care. CMAJ 2010;182:E839–42. 10.1503/cmaj.090449 20603348 PMC3001530

[47] Ben Abdeljelil A , Freire GC , Yanchar N , et al . Pediatric moderate and severe traumatic brain injury: a systematic review of clinical practice guideline recommendations. J Neurotrauma 2023;40:2270–81. 10.1089/neu.2023.0149 37341019

[48] Freire GC , Beno S , Yanchar N , et al . Clinical practice guideline recommendations for pediatric multisystem trauma care: a systematic review. Annals of Surgery 2023;278:858–64. 10.1097/SLA.0000000000005966 37325908

[49] Yanchar N , Tardif P-A , Freire G , et al . Clinical practice guidelines recommendations for pediatric solid organ injury care: a systematic review. J Trauma Acute Care Surg 2023;95:442–50. 10.1097/TA.0000000000004015 37272747

[50] Moberg J , Oxman AD , Rosenbaum S , et al . The GRADE evidence to decision (Etd) framework for health system and public health decisions. Health Res Policy Syst 2018;16:45. 10.1186/s12961-018-0320-2 29843743 PMC5975536

[51] Lunny C , Salzwedel DM , Liu T , et al . Validation of five search filters for retrieval of clinical practice guidelines produced low precision. J Clin Epidemiol 2020;117:109–16. 10.1016/j.jclinepi.2019.09.022 31610216

