# Supplementary material for: Clinical practice guideline recommendations to improve the mental health of adult trauma patients: protocol for a systematic review
Source: BMJ Open. 2024 Mar 25;14(3):e079205. doi: 10.1136/bmjopen-2023-079205 (PMC10966799; doi:10.1136/bmjopen-2023-079205)
Supplement: Author's manuscript [file bmjopen-2023-079205.draft_revisions.pdf]

BMJ Open

BMJ Open is committed to open peer review. As part of this commitment we make the peer review history of every article we publish publicly available.

When an article is published we post the peer reviewers' comments and the authors' responses online. We also post the versions of the paper that were used during peer review. These are the versions that the peer review comments apply to.

The versions of the paper that follow are the versions that were submitted during the peer review process. They are not the versions of record or the final published versions. They should not be cited or distributed as the published version of this manuscript.

BMJ Open is an open access journal and the full, final, typeset and author-corrected version of record of the manuscript is available on our site with no access controls, subscription charges or pay-per-view fees (http://bmjopen.bmj.com).

If you have any questions on BMJ Open's open peer review process please email info.bmjopen@bmj.com

BMJ Open

Clinical practice guideline recommendations to improve the mental health of adult trauma patients: protocol for a systematic review

Journal:	*BMJ Open*
Manuscript ID	bmjopen-2023-079205
Article Type:	Protocol
Date Submitted by the Author:	24-Aug-2023
Complete List of Authors:	Berube, Melanie; CHU de Quebec-Universite Laval, ; Universite Laval, Faculty of Nursing<br>Bradley, Nori; University of Alberta Hospital<br>O'Donnell, Meaghan; The University of Melbourne Faculty of Medicine Dentistry and Health Sciences, Department of Psychiatry<br>Stelfox, Henry; University of Calgary, Critical Care Medicine<br>Garraway, Naisan; Vancouver General Hospital<br>Vasiliadis, Helen-Maria; University of Sherbrooke<br>Turcotte, Valerie; Université Laval<br>Perreault , M; Douglas Mental Health University Institute,<br>Menear, Matthew; Universite Laval, Family Medicine and Emergency Medicine<br>Archambault, Leonie; Institut universitaire en santé mentale Douglas<br>Haagsma, Juanita; Erasmus MC, Public Health<br>Provencher, Helene; Université Laval<br>Genest, Christine; Université de Montréal, Faculté des sciences infirmières; Centre de recherche de l'Institut universitaire en sante mentale de Montreal,<br>Gagnon, Marc-Aurèle; Centre de recherche du CHU de Québec-Université Laval, Population Health and Optimal Practices<br>Bourque, Laurence; CHU de Quebec-Universite Laval, Population Health and Optimal Practices Research Unit Research Unit<br>Lapierre, Alexandra; Hôpital du Sacré-Coeur de Montréal Centre de Recherche,<br>Amal, Kalfi; Université Laval<br>Panenka, William; The University of British Columbia
Keywords:	TRAUMA MANAGEMENT, MENTAL HEALTH, Protocols & guidelines < HEALTH SERVICES ADMINISTRATION & MANAGEMENT, Systematic Review

SCHOLARONE™
Manuscripts

Clinical practice guideline recommendations to improve the mental health of adult trauma patients: protocol for a systematic review

Mélanie Bérubé[1,2], Nori Bradley[3,4], Meaghan O'Donnell[5], Henry Thomas Stelfox[6], Naisan Garraway[7], Helen Maria Vasiliadis[8], Valérie Turcotte[2,9], Michel Perreault[10,11], Matthew Menear[12,13], Léonie Archambault[11], Juanita Haagsma[14], Hélène Provencher[2], Christine Genest[15], Marc-Aurèle Gagnon[1], Laurence Bourque[1], Alexandra Lapierre[1], Amal Khalfi[1,2], William Panenka[16,17]

[1]CHU de Québec-Université Laval Research Centre, Population Health and Optimal Practices Research Unit Research Unit (Trauma – Emergency – Critical Care Medicine), 1401, 18e rue, Québec City, Quebec, Canada, GIV 1Z4

[2]Faculty of Nursing, 1050 Avenue de la Médecine, Université Laval, Québec City, Quebec, Canada, G1V 0A6

[3]University of Albert Hospital, 8440 112 St NW, Edmonton, Alberta, Canada, T6G 2b7

[4]Royal Columbian Hospital, 330E Columbia St, New Westminster, British Columbia, V3L 3W7

[5]University of Melbourne, Department of Psychiatry, 161 Barry Street, Carlton VIC 30150

[6]University of Calgary & Alberta Health Services, O'Brien Institute for Public Health, 3280 Hospital Dr NW, Calgary, Alberta, Canada, T2N 4Z6

[7]Vancouver General Hospital, Trauma Services, 855 West 12 Avenue, Vancouver, British Columbia, Canada, V5Z 1M9

[8]Université de Sherbrooke, Community Health Sciences, 150 Place Charles Lemoyne, Longueil, Quebec, Canada, J4K 0A8

[9]CIUSSS du Nord-de-l'île-de-Montréal, Department of Nursing, 5400 Boulevard Gouin Ouest Montréal, Quebec, Canada, H4J 1C5

[10]McGill University, Department of Psychiatry, 1033 Pine Ave W, Montreal, Quebec, Canada, H3A 1A1

[11]Institut universitaire en santé mentale Douglas, 6875 Bd LaSalle, Verdun, Quebec, Canada, H4H 1R3

[12]Université Laval, Department of Family and Emergency Medicine, 1050 Avenue de la Médecine, Université Laval, Québec City, Quebec, Canada, G1V 0A6

[13]Centre de recherche en santé durable, Centre intégré universitaire de santé et de services sociaux de la Capitale-Nationale, 2480, chemin de la Canardière, Québec, Quebec, Canada, G1J 2G1

[14]Erasmus University, Department of Public Health, Dr. Molewaterplein 40, 3015 GD Rotterdam, Netherlands.

[15]Université de Montréal, Faculty of Nursing, 2375, chemin de la Côte-Ste-Catherine, Montreal, Quebec, Canada, H3T 1A8

[16]University of British Columbia, Department of Psychiatry, 2255 Wesbrook Mall, Vancouver, British Columbia, Canada, V6T 2A1

Corresponding author:
Mélanie Bérubé
CHU de Québec-Université Laval Research Centre
1401, 18e rue, Québec City, Quebec, Canada, GIV 1Z4
T 418 649-0252 ext: 66600
email: melanie.berube@fsi.ulaval.ca

For peer review only - http://bmjopen.bmj.com/site/about/guidelines.xhtml

Abstract

Introduction: Mental disorders are common in adult patients with traumatic injuries. To limit the burden of poor psychological well-being in this population, recognized authorities have issued recommendations through clinical practice guidelines (CPGs). However, the uptake of evidence-based recommendations to improve the mental health of trauma patients is low. This may be explained by the difficulty healthcare professionals have in interpretating CGPs scope and quality. Our aim is to systematically review CPG mental health recommendations in the context of trauma care and appraise their quality.

Methods and analysis: We will identify CPG recommendations through a search strategy applied to Medline, Embase, CINAHL, PsycINFO and Web of Science databases, as well as guidelines repositories and websites of trauma associations. We will target CPGs on adult (≥ 18 years old) and acute trauma populations including at least one recommendation on any prevention, screening, assessment, intervention, patient and family engagement, referral, or follow-up procedure related to mental health with any comparator endorsed by recognized organizations in high-income countries. No language limitations will be applied, but we will limit the search to the last 15 years. Pairs of reviewers will independently screen titles, abstracts, full texts, and carry out data extraction and quality assessment of CPGs and their recommendations using the Appraisal of Guidelines Research and Evaluation (AGREE) II. We will synthesize the evidence on recommendations for CPGs rated as moderate or high quality using a recommendation matrix based on the GRADE quality of evidence, strength of recommendation, and health and social determinants.

Ethics and dissemination: Ethics approval is not required, as we will conduct secondary analysis of published data. The results will be disseminated in a peer-reviewed journal, at international and national scientific meetings, and an accessible summary will be distributed to interested parties through professional, healthcare quality and persons with lived experience associations.

PROSPERO registration number : International Prospective Register of Systematic Reviews (ID454728)

Keywords: trauma care, mental health, best practices, implementation science

Strengths and limitations of this study

- We will produce a meta-synthesis of clinical practice guidelines (CPGs) recommendations on mental health in injury care based on their methodological quality and level of evidence.
- Our review involves interested parties to maximize its relevance and reach.
- We will focus solely on CPGs for mental health from high-income countries.
- Our search strategy is not designed to identify CPGs that do not specifically focus on mental health in trauma populations.

For peer review only - http://bmjopen.bmj.com/site/about/guidelines.xhtml

Introduction

Improvements in trauma care in high-income countries over the past 30 years have reduced mortality from as high as 50% to 11%.[1,2] As a result, more and more patients survive their traumatic injuries, but still face a significant burden of morbidity. Indeed, traumatic injuries are a leading cause of disability[3,4], and result in the highest number of productive years of life lost.[5,6]

The impact of traumatic injuries is not without consequences for patients' mental health, not to mention the fact that many are already vulnerable on a psychosocial level. Before the injury, 20-40% of trauma patients have mental health issues, including substance use disorders and mood disorders[7,8], which can impair recovery.[9] After the injury, up to 45% of patients develop acute stress disorder during their hospital stay[10], and 10-50% report post-traumatic stress disorder (PTSD), the prevalence varying according to the phases of their recovery and trauma mechanisms.[9, 11-15] Hence, patients who sustained traumatic injuries have a 40% higher risk of being hospitalized for a mental disorder when comparing the five years before and after the injury.[16] Cumulative structural, social, and health determinants have been shown to contribute significantly to mental disorders among trauma patients with racial/ethnic minorities, those with low levels of education and financial resources, males or patients identifying themselves as men, and those living in rural areas being most vulnerable.[16-18]

The high prevalence of mental disorders in trauma patients is alarming considering that they have profound negative impacts on individual and social outcomes. Trauma patients suffering from mental disorders were shown to have twice as many complications and twice the length of hospital stays.[19] Similarly, these patients are almost 10 times more likely to develop chronic pain and have physical limitations, and two to four times more likely not to return to work than patients without mental disorders[20], while having a poorer quality of life.[14]

For peer review only - http://bmjopen.bmj.com/site/about/guidelines.xhtml

Given the growing body of research evidence highlighting the burden of mental health issues following traumatic injury, recognized authorities[21-24] have proposed clinical practice guidelines (CPGs) to reduce their prevalence. CPG recommendations apply to all healthcare professionals (HCPs) involved in the trauma care continuum, and cover mental health promotion/illness prevention, screening, brief and more comprehensive interventions, patient referral to subsequent specialty services and follow-up, and patient and family centered-care approach (i.e., collaborative approach, share-decision making[25]). However, HCPs' adherence rates to CPGs are reported to be less than 30%.[26,27] Lack of knowledge about evidence-based recommendations, as well as the capacity and time required to assess their scope and quality, may be at the root of this low uptake rate.[28] Hence, we aim to systematically review CPG recommendations for mental health in the context of injury care and appraise their quality.

Methods

In line with the knowledge synthesis phase of the Knowledge to Action framework[29], we will conduct a knowledge synthesis on CPGs. This protocol was developed according to Cochrane recommendations for systematic reviews (SR)[30] and methodological guidelines for systematic review on CPGs[31] and is reported according to the Preferred Reporting Items for Systematic Review and Meta-Analysis Protocol statement.[32]

Patient and public involvement

To maximize contributions, we set up an advisory committee including representatives of associations that play a role in setting standards for trauma and mental health care (Trauma Association of Canada, the Mental Health Commission of Canada, and the *Institut national d'excellence en santé et en services sociaux*) and in supporting persons with lived experience (Brain Injury Canada, Mothers Against Drunk Driving - Canada, *Moelle épinière et motricité Québec, and Connexion TCC-Québec*). The advisory committee also includes HCPs from various disciplines involved with trauma patients (specialized physicians/surgeons, nurses, rehabilitation professionals, psychologists, social workers), persons with

For peer review only - http://bmjopen.bmj.com/site/about/guidelines.xhtml

lived experience, and decision makers. The committee was involved in the development of the protocol and will oversee the progress of the review and take part in disseminating the results.

Eligibility

We will target CPGs on adult (≥ 18 years old) and in-patient acute trauma populations (P) including at least one recommendation (R) on any prevention, screening, assessment, intervention, patient and family engagement, referral, or follow-up procedure related to mental health (I) with any or no comparator (C) endorsed by recognized organizations in high-income countries within the last 15 years (September 2008 to a maximum of six months prior to submission) (A). No language restriction will be applied. We will use an open-access online translator (https://www.deepl.com/translator) for studies not written in English of French.[33] CGPs are defined as "statements that include recommendations intended to optimize patient care that are informed by a review of evidence and an assessment of benefits and harms of alternative care options".[34] We will include CPGs focusing on the categories of mental disorders defined in the Diagnostic and Statistical Manual of Mental Disorders fifth edition-text revision (DSM-5-TR)[35] that most often affect trauma patients: trauma-and-stressor-related disorders, anxiety disorders, substance-related and addictive disorders, suicidal behavior disorder and self-injury.[7,8,16] High-income countries, based on World Bank definitions[36], will be targeted to ensure that recommendations are compatible with the practices of accredited trauma centers[37], while the time limit aims to focus on recent recommendations.

Search strategy

Based on the recommendations of the Peer Review of Electronic Search Strategies[38], we will establish a systematic search strategy in collaboration with an information specialist, which will be independently reviewed by a second information specialist. We will use a combination of controlled terms (e.g., MeSH for MEDLINE, Emtree for Embase) and free vocabulary according to the following themes: wounds and injuries, trauma, mental health, mental disorders, guidelines. We will apply the pilot search strategy in Medline (Ovid) and Embase (Ovid) databases. Relevant words found in the title, abstract and text of retrieved studies will be collected. We will then add these words and indexing terms to the search

For peer review only - http://bmjopen.bmj.com/site/about/guidelines.xhtml

strategy, which will be executed in the Medline (Ovid), Embase (Ovid), CINAHL (EBSCO), PsycINFO (Ovid) and Web of Science (Clarivate) databases. Relevant GPGs will also be searched in CPGs repositories and trauma association websites, a list of which was drawn up with members of the research team who have in-depth knowledge of the innerworkings in this field. Using a preliminary search strategy (from January 2018 to August 8th 2023; table 1 and table 2), we have identified 3882 citations, including 5 sentinel GPCs[21,23,24,39,40] identified *a priori*, indicating good sensitivity and specificity.

CPGs selection and data extraction

We will manage citations using EndNote (version X9.3.3, New York City: Thomson Reuters, 2018). Pairs of reviewers will independently screen titles, abstracts and full-texts, and carry out data extraction. Once the final set of included CPGs has been obtained, all associated methodological supplements will be retrieved before data extraction or quality assessment begins.[31] Data from included CPGs will be extracted with an extraction form, which will be pilot-tested on five items to ensure feasibility and completeness.[30] For each recommendation within CPGs, we will extract information on the characteristics of included CPGs: title and year, country, organization, target users, population including determinants contributing to disparities in mental health care[41,42] (e.g., type of trauma, gender, age group, ethnocultural group, geographical location, socioeconomic status), areas of focus, intervention, quality of evidence and strength of recommendations. If important information is missing or unclear, we will request it by sending up to three emails to the first, second and last authors.

Quality

Pairs of reviewers with content expertise will independently assess CPGs quality based on the six domains of the AGREE II tool[43]: 1) scope and purpose (overall aim of the guideline, specific health questions, and target population), 2) interested parties involvement (developed by the appropriate stakeholders, consistent with the views of its intended users), 3) rigorously developed (process used to gather and synthesize the evidence, methods to formulate and update the recommendations), 4) clarity and presentation (language, structure, and format), 5) applicability (barriers and facilitators to

For peer review only - http://bmjopen.bmj.com/site/about/guidelines.xhtml

implementation, strategies to improve uptake, demand on healthcare resources), and 6) editorial independence (competing interests reported and addressed). In accordance with guidelines on the systematic review of CPGs[31] and based on previous systematic reviews of CPGs[44-46], each domain with a score ≥60% will be considered effectively addressed. CPGs will be considered *high quality* if they score ≥60% in at least three of the six AGREE II domains, including domain 3 (rigor of development). If three domains or more score ≥60%, and domain 3 score <60%, the CPG will be considered *moderate quality*. CPGs scoring <60% in two domains or more and scoring <50% in domain 3 will be considered *low quality*.

Data synthesis

CPG recommendations will be classified into the following key areas, as determined by the advisory committee: 1) mental health promotion/illness prevention, 2) screening for mental disorders, 3) assessment, 4) interventions, 5) referral for patients experiencing mental disorders for follow-up and support services, and 6) patient and family centered-care approach. We will synthesize the evidence on recommendations for CPGs rated as moderate or high quality (AGREE II)[47] using a recommendation matrix based on the GRADE quality of evidence (high, moderate, low and very low), recommendation strength (low, high)[44-46], and health and social determinants (i.e., care equity). Matrix data will be extracted independently by pairs or reviewers for each recommendation. We will group the same or very similar recommendations published by more than one CPG. For CPGs that used a rating system other than GRADE, we will match reported categories to GRADE categories.

For selection, extraction, quality assessment and meta-synthesis, all reviewers will systematically and iteratively pilot test the process on a random selection of articles (200 citations for selection and 5 CPGs for extraction and quality evaluation and meta-synthesis in each iteration) until acceptable agreement is achieved (kappa>0.8).[30] Any discrepancies during the review process will be resolved by first consulting a senior member of the research team (WP), and then by reaching a consensus among the members of the advisory committee, if necessary.

For peer review only - http://bmjopen.bmj.com/site/about/guidelines.xhtml

Limitation of study

For feasibility reasons, our search strategy was not developed to systematically identify CPGs that do not specifically target mental disorders in trauma populations. Therefore, we may miss recommendations on mental health if they are included in CPGs that target trauma populations in general if no keywords relating to mental health and injury are present in the title or abstract. However, these recommendations are likely to be identified by consulting CPGs repositories and trauma association websites (Table 2), which were recognized as the best sources to find relevant guidelines.[48]

Potential impact

Recent data on mental health issues in trauma populations are now prompting recognized authorities[21-24] to prioritize this area to further improve the outcomes in injured patients. However, to date, there has been no synthesis of recommendations for improving mental health practices in the trauma field. This systematic review of CPGs will allow to identify recommendations with the greatest support for implementation, thus taking into account the staff shortage and time constraints omnipresent in healthcare systems. Results may be used by healthcare professionals, trauma program leaders, hospital administrators and policymakers to inform local quality improvement initiatives, identify system-wide problems and plan future actions to improve mental health practices.

Ethics and dissemination

Research ethics approval is not required, as we will conduct secondary analysis of published data. The results of our study will be disseminated in a peer-reviewed journal, at international and national scientific meetings, and an accessible summary will be distributed to stakeholders through professional, healthcare quality and persons with lived experience associations.

Twitter: @BrubMlanie1, @BradleyNori, @Turcottetrauma, @Alexe_Lapierre

Contributors : All authors were involved in conceiving and designing the protocol and read, revised and approved the final manuscript. MB and LB drafted the manuscript.

For peer review only

For peer review only - http://bmjopen.bmj.com/site/about/guidelines.xhtml

Funding: This work is supported by the Fonds de Recherche en Santé du Québec and the Strategy for Patient-Oriented Research-Quebec and the Quebec Nursing Intervention Network. Funders had not role in developing the protocol.

Competing interests: None to declare

Patient and public involvement: Patients and/or the public were not involved in the design, or conduct, or reporting, or dissemination plans of this research.

Provenance and peer reviewed: Not commissioned; externally peer reviewed.

For peer review only

References

1. Moore L, Evans D, Hameed SM, *et al*. Mortality in Canadian Trauma Systems: A Multicenter Cohort Study. Ann Surg 2017; **265**(1): 212-17. doi: 10.1097/sla.0000000000001614
2. Truchon C, Moore L, Belcaid A, *et al*. Shaping Quality Through Vision, Structure, and Monitoring of Performance and Quality Indicators: Impact Story From the Quebec Trauma Network. Int J Technol Assess Health Care 2017;**33**(4):415-9. doi: 10.1017/s0266462317000198
3. World Health Organization. WHO reveals leading causes of death and disability worldwide: 2000-2019, 2020. Available: https://www.who.int/news/item/09-12-2020-who-reveals-leading-causes-of-death-and-disability-worldwide-2000-2019
4. GBD 2019 Diseases and Injuries Collaborators. Global burden of 369 diseases and injuries in 204 countries and territories, 1990–2019: a systematic analysis for the Global Burden of Disease Study 2019. Lancet 2020;**396**:1204-22. doi: 10.1016/S0140-6736(20)30925-9
5. Parachute. Cost of Injury in Canada, 2022. Available: https://parachute.ca/en/professional-resource/cost-of-injury-in-canada/
6. Centers for Disease Control and Prevention. Injury Prevention & Control: Cost of Injury Data, 2021. Available: https://www.cdc.gov/injury/wisqars/cost/
7. Clous EA, Beerthuizen KC, Ponsen KJ, *et al*. Trauma and psychiatric disorders: A systematic review. J Trauma Acute Care Surg 2017;**82**(4):794-801. doi: 10.1097/TA.0000000000001371.
8. Weinberg DS, Narayanan AS, Boden KA, *et al*. Psychiatric Illness Is Common Among Patients with Orthopaedic Polytrauma and Is Linked with Poor Outcomes. J Bone Joint Surg Am 2016;**98**(5):341-8. doi: 10.2106/JBJS.15.00751
9. Kruithof N, Polinder S, de Munter L, *et al*. Health status and psychological outcomes after trauma: A prospective multicenter cohort study. PLoS One 2020;**15**(4):e0231649. doi: 10.1371/journal.pone.0231649. eCollection 2020.
10. Wiseman T, Foster K, Curtis K. Mental health following traumatic physical injury: an integrative literature review. Injury 2013; **44**(11):1383-90. doi: 10.1016/j.injury.2012.02.015. Epub 2012 Mar 10.
11. Lin W, Gong L, Xia M, Dai W. Prevalence of posttraumatic stress disorder among road traffic accident survivors: A PRISMA-compliant meta-analysis. Medicine (Baltimore) 2018;**97**(3):e9693. doi: 10.1097/MD.0000000000009693.
12. Roden-Foreman K, Solis J, Jones A, *et al*. Prospective Evaluation of Posttraumatic Stress Disorder and Depression in Orthopaedic Injury Patients With and Without Concomitant Traumatic Brain Injury. J Orthop Trauma 2017;**31**(9):e275-e80. doi: 10.1097/BOT.0000000000000884.
13. Joseph NM, Benedick A, Flanagan CD, *et al*. Risk Factors for Posttraumatic Stress Disorder in Acute Trauma Patients. J Orthop Trauma 2021;**35**(6):e209-e15. doi: 10.1097/BOT.0000000000001990.
14. van der Vlegel M, Polinder S, Toet H, *et al*. Anxiety, depression and post-traumatic stress symptoms among injury patients and the association with outcome after injury. Eur J Psychotraumatol 2022;**13**(1):2023422. doi: 10.1080/20008198.2021.2023422. eCollection 2022.
15. Warren AM, Jones AL, Bennett M, *et al*. Prospective Evaluation of Posttraumatic Stress Disorder in Injured Patients With and Without Orthopaedic Injury. J Orthop Trauma 2016;**30**(9):e305-11. doi: 10.1097/BOT.0000000000000623.
16. Evans CCD, DeWit Y, Seitz D, *et al*. Mental health outcomes after major trauma in Ontario: a population-based analysis. Cmaj 2018;**190**(45):E1319-e27. doi: 10.1503/cmaj.180368.

For peer review only - http://bmjopen.bmj.com/site/about/guidelines.xhtml

17. Herrera-Escobar JP, Seshadri AJ, Stanek E, *et al*. Mental Health Burden After Injury: It's About More than Just Posttraumatic Stress Disorder. Ann Surg 2021;**274**(6):e1162-e9. doi: 10.1097/SLA.0000000000003780
18 Mollayeva T, D'Souza A, Mollayeva S. Systematic Review of Sex and Gender Effects in Traumatic Brain Injury: Equity in Clinical and Functional Outcomes. Front Neurol 2021;**12**:678971. doi: 10.3389/fneur.2021.678971. eCollection 2021
19. Sharwood LN, Wiseman T, Tseris E, *et al*. Pre-existing mental disorder, clinical profile, inpatient services and costs in people hospitalised following traumatic spinal injury: a whole population record linkage study. Inj Prev 2020. doi: 10.1136/injuryprev-2019-043567
20. Herrera-Escobar JP, Al Rafai SS, Seshadri AJ, *et al*. A multicenter study of post-traumatic stress disorder after injury: Mechanism matters more than injury severity. Surgery 2018;**164**(6):1246-50. doi: 10.1016/j.surg.2018.07.017. Epub 2018 Aug 29.
21. Committee on Trauma-American College of Surgeons. Best Practices Guidelines: Screening and Interventions for Mental Health Disorders and Subctance Use in the Acute Trauma Patients, 2022. Available: https://www.facs.org/quality-programs/trauma/quality/best-practices-guidelines/
22. Substance Abuse and Mental Health Services Administration. Substance Use Disorder Treatment for People With Physical and Cognitive Disabilities Advisory, 2019. Available: https://store.samhsa.gov/sites/default/files/d7/priv/pep19-02-00002_508_022620.pdf
23. Bombardier CH, Azuero CB, Fann JR, *et al*. Management of Mental Health Disorders, Substance Use Disorders, and Suicide in Adults with Spinal Cord Injury: Clinical Practice Guideline for Healthcare Providers. Top Spinal Cord Inj Rehabil 2021;**27**(2):152-224. doi: 10.46292/sci2702-152
24. Major Extremity trauma and Rehabilitation Consortium and the American Academy of Orthopaedic Surgeons. Evaluation of Psychosocial Factors Influencing Recovery from Adult Orthopeadic Trauma, 2019. Available: https://www.aaos.org/globalassets/quality-and-practice-resources/dod/prf-cpg-final-draft-12-3-19.pdf
25. World Health Organization. WHO global strategy on people-centred and integrated health services, 2015. Available: https://interprofessional.global/wp-content/uploads/2019/11/WHO-2015-Global-strategy-on-integrated-people-centred-health-services-2016-2026.pdf
26. Lamontagne ME, Gargaro J, Marier-Deschênes P, *et al.* A Survey of Perceived Implementation Gaps for a Clinical Practice Guideline for the Rehabilitation of Adults With Moderate to Severe Traumatic Brain Injury. J Head Trauma Rehabil 2018;**33**(5):306-16. doi: 10.1097/HTR.0000000000000430
27. Bulger EM, Johnson P, Parker L, *et al*. Nationwide Survey of Trauma Center Screening and Intervention Practices for Posttraumatic Stress Disorder, Firearm Violence, Mental Health, and Substance Use Disorders. J Am Coll Surg 2022;**234**(3):274-87. doi: 10.1097/XCS.0000000000000064
28. Lamontagne ME, Bayley MT, Marshall S, *et al*. Assessment of Users' Needs and Expectations Toward Clinical Practice Guidelines to Support the Rehabilitation of Adults With Moderate to Severe Traumatic Brain Injury. J Head Trauma Rehabil 2018;**33**(5):288-95. doi: 10.1097/HTR.0000000000000429
29. Graham ID, Logan J, Harrison MB, *et al*. Lost in knowledge translation: time for a map? J Contin Educ Health Prof 2006;**26**(1):13-24. doi: 10.1002/chp.47
30. Higgins J TJ, Chandler J, Cumpston M, Li T, *et al*. Cochrane Handbook for Systematic Reviews of Interventions version 6.3, 2022. Available: www.training.cochrane.org/handbook
31. Johnston A, Kelly SE, Hsieh SC, *et al*. Systematic reviews of clinical practice guidelines: a methodological guide. J Clin Epidemiol 2019;**108**:64-76. doi: 10.1016/j.jclinepi.2018.11.030. Epub 2018 Dec 5.

For peer review only - http://bmjopen.bmj.com/site/about/guidelines.xhtml

32. Moher D, Shamseer L, Clarke M, *et al*. Preferred reporting items for systematic review and meta-analysis protocols (PRISMA-P) 2015 statement. Systematic Reviews 2015;**4**(1):1. doi: 10.1186/2046-4053-4-1
33. Takakusagi Y, Oike T, Shirai K, *et al*. Validation of the Reliability of Machine Translation for a Medical Article From Japanese to English Using DeepL Translator. Cureus 2021; **13**(9): e17778. doi: 10.7759/cureus.17778. eCollection 2021 Sep.
34. Institute of Medicine (US) Committee on Standards for Developing Trustworthy Clinical Practice Guidelines, *Clinical Practice Guidelines We Can Trust*, ed. R. Graham, M. Mancher, and W.D. Miller. 2011: Washington (DC): National Academies Press (US).
35. American Psychiatric Association. Diagnostic and Statistical Manual of Mental Disorders. Fifth Edition, Text-Revision (DSM-TR), 2022: Washington (DC), US.
36. Blogs WB. New World Bank country classifications by income level: 2022-2023. [cited 2023 August 20th]. Available: https://blogs.worldbank.org/opendata/new-world-bank-country-classifications-income-level-2022-2023
37. Committee on Trauma-American College of Surgeons. Resources for Optimal Care of the Injured Patient, 2022. Available: https://www.facs.org/quality-programs/trauma/quality/verification-review-and-consultation-program/standards/
38. McGowan J, Sampson M, Salzwedel DM, *et al*. PRESS Peer Review of Electronic Search Strategies: 2015 Guideline Statement. J Clin Epidemiol 2016;**75**:40-6. doi: 10.1016/j.jclinepi.2016.01.021
39. Metha S OS, Blackport D, Teasell R. Mental Health after a Spinal Cord Injury, 2022. Available: https://scireproject.com/evidence/mental-health/introduction/
40. Kodadek LM, Freeman JJ, Tiwary D, *et al*. Alcohol-related trauma reinjury prevention with hospital-based screening in adult populations: An Eastern Association for the Surgery of Trauma evidence-based systematic review. J Trauma Acute Care Surg 2020;**88**(1):106-12. doi: 10.1097/ta.0000000000002501
41. Haider AH, Weygandt PL, Bentley JM, *et al*. Disparities in trauma care and outcomes in the. United States: a. systematic review and meta-analysis. J. Trauma Acute care Surg 2013;7**4**(5);1195-205. doi: 10.1097/TA.0b013e31828c331d
42. Englum BR, Villegas C, Bolorunduro O, *et al*. Racial, ethnic, and insurance status disparities in use of posthospitalization care after trauma. J Am Coll Surg 2011;**213**(6):699-708. doi: 10.1016/j.jamcollsurg.2011.08.017
43. Brouwers MC, Kho ME, Browman GP, *et al*. AGREE II: advancing guideline development, reporting and evaluation in health care. Cmaj 2010;**182**(18):E839-42. doi: 10.1503/cmaj.090449
44. Ben Abdeljelil A, Freire GC, Yanchar N, *et al*. Pediatric Moderate and Severe Traumatic Brain Injury: A Systematic Review of Clinical Practice Guideline Recommendations. J Neurotrauma 2023 (ahead of print). doi: 10.1089/neu.2023.0149
45. Freire GC, Beno S, Yanchar N, *et al*. Clinical Practice Guideline Recommendations For Pediatric Multisystem Trauma Care: A Systematic Review. Ann Surg 2023 (ahead of print). doi: 10.1097/SLA.0000000000005966
46. Yanchar N, Tardif PA, Freire G, e*t al*. Clinical Practice Guidelines Recommendations for Pediatric Solid Organ Injury Care: A Systematic Review. J Trauma Acute Care Surg 2023 (ahead of print). doi: 10.1097/TA.0000000000004015
47. Moberg J, Oxman AD, Rosenbaum S, *et al*. The GRADE Evidence to Decision (EtD) framework for health system and public health decisions. Health Res Policy Syst 2018;**16**(1):45. doi: 10.1186/s12961-018-0320-2
48. Lunny C, Salzwedel DM, Liu T, *et al*. Validation of five search filters for retrieval of clinical practice guidelines produced low precision. J Clin Epidemiol 2020;**117**:109-16. doi: 10.1016/j.jclinepi.2019.09.022

For peer review only - http://bmjopen.bmj.com/site/about/guidelines.xhtml

Table 1. Medline (Ovid) (August 8th, 2023)

Concepts	Search strategy keywords	Research and no of results
**Trauma/ recovery** (Controlled vocabulary)	exp "Wounds and Injuries"/ or exp Brain Hemorrhage, Traumatic/ or Brain Injuries/ or Coma, Post-Head Injury/ or Craniocerebral Trauma/ or Diffuse Axonal Injury/ or exp Fractures, Bone/ or Head Injuries, Closed/ or Head Injuries, Penetrating/ or exp Intracranial Hemorrhage, Traumatic/ or exp Skull Fractures/	#1<br>1 007 620
	Fractur* OR Injur* OR TBI OR trauma*	#2<br>1 430 550
(Free text)	1 OR 2	#3<br>1 894 323
**Mental health** (Controlled vocabulary)	exp Mental Disorders/ or exp Mental Health/ or exp Substance-Related Disorders/	#4<br>1 501 202
(Free text)	"mental health" OR "mental disorder*" OR "mental illness" OR "post-traumatic stress disorder" OR "post traumatic stress disorder" OR "PTSD" OR "Acute stress disorder*" or "acute stress reaction*" or "adjustment disorder*" or "suicidal behavior*" or "self-injur*" or "self injur*" or "self-harm" or "self harm" or "depressive disorder*" or "depression" or "mood disorder*" or "anxiety disorder*" or "trauma and stressor-related disorder*" or "trauma and stressor related disorder*" or "Mental distress" or "psychological distress" OR ((drug* OR substance OR alcohol OR marijuana OR cannabis OR narcotic* OR opiate* OR opioid* OR opium) Adj4 (abus* OR "use" OR user OR usage OR misus* OR usin* OR utilis* OR depend* OR addict* OR illegal* OR illicit* OR habit* OR withdraw* OR behavi* OR abstinence* OR abstain* OR intoxica* OR addict* OR disorder*)) OR "drug rehabilitation" OR "non-prescription drugs" OR "non-prescription drug"	#5<br>1 069 467
	4 OR 5	#6<br>2 121 781
**Guideline** (Controlled vocabulary)	exp Practice Guidelines as Topic/ or exp Practice Guideline/ or exp Guidelines as Topic/ or exp Guideline/	#7<br>208 566
(Free text)	Guide OR Guideline*	#8<br>676 237
	7 OR 8	#9<br>784 520
**Total**	3 AND 6 AND 9<br>Limit to 2008-2023	3 882

For peer review only

For peer review only - http://bmjopen.bmj.com/site/about/guidelines.xhtml

Table 2. Preliminary list of clinical practice guidelines repositories and trauma professional associations

1-Agency for Healthcare Research and Quality	32-Guideline Central
2-Accreditation Canada	33- Guidelines International Network
3- Australasian Association for Quality in Healthcare	34-Headway—the brain injury association
4-American Academy of Orthopedic Surgeons	35- International Association for Trauma Surgery and Intensive Care
5-American Association for the Surgery on Trauma	36-International Society of Orthopaedic and Traumatology
6- American Association of Neurological Surgeons	37-Italian Society of Orthopaedics and Traumatology
7- American Board of Orthopedic Surgery	38- National Association for Healthcare Quality
8-American College of Surgeons	39-National Guidelines Clearinghouse
9-American Orthopaedic Association	40- National Institute of Health and Care Excellence
10-American Spinal Injury Association	41- National Quality Forum
11-American Trauma Society	42-Norwegian Neurosurgical Association
12-Australian Society of Orthopeadic Surgeons	43-Ontario Neurotrauma Foundation
13- Australian and New Zealand Society	44- Orthopedic Trauma Association
14-Belgian Society for Orthopedics and Traumatology	45-Scandinavian Neurotrauma Committee
15-Brain Injury Association of America	46-Scottish Intercollegiate Guidelines Network
16-Brain Trauma Foundation	47-Société Française de Chirurgie Orthopédique et Traumatologique
17- British Orthopaedic Association	48-Society of Critical Care Medicine Trauma Network
18-British Trauma Society	49-Société Royale Belge de Chirurgie Orthopédique et de Traumatologie
19- Canada's Drug and Health Technology Agency	50-Spanish Society of Orthopaedic Surgery and Traumatology
20-Centers for Disease Control and Prevention	51- Society of Trauma Nurses
21-Congress of Neurological Surgeons	52-Spinal Cord Injury Research Evidence
22- Consortium of Spinal Cord Medicine	53-Sustance Abuse and Mental Health Services Administrations

For peer review only - http://bmjopen.bmj.com/site/about/guidelines.xhtml

23-Danish Neurosurgical Society	54-Swedish Neurosurgical Societ
24-Dutch Institute for Healthcare Improvement	55-Trauma Association of Canada
25- Eastern Association for the Surgery of Trauma	56-Trauma Audit Research Network
26-European Association of Neurosurgical Societies	57-Trauma.org
27-European Federations of National Associations of Orthopaedic and Traumatology	58-U.S. Department of Health and Human Services
28-European Society for Trauma and Emergency Surgery	59-U.S. Department of Veterans Affairs
29-Finnish Association of Neuroscience Nurses	60-Western Trauma Association
30-Finnish Neurosurgical Society	61-World Health Organization
31-Greek Society of Orthopaedic and Traumatological Surgery	

For peer review only

BMJ Open

Clinical practice guideline recommendations to improve the mental health of adult trauma patients: protocol for a systematic review

Journal:	*BMJ Open*
Manuscript ID	bmjopen-2023-079205.R1
Article Type:	Protocol
Date Submitted by the Author:	20-Feb-2024
Complete List of Authors:	Berube, Melanie; CHU de Quebec-Universite Laval, ; Universite Laval, Faculty of Nursing<br>Bradley, Nori; University of Alberta Hospital<br>O'Donnell, Meaghan; The University of Melbourne Faculty of Medicine Dentistry and Health Sciences, Department of Psychiatry<br>Stelfox, Henry; University of Calgary, Critical Care Medicine<br>Garraway, Naisan; Vancouver General Hospital<br>Vasiliadis, Helen-Maria; University of Sherbrooke<br>Turcotte, Valerie; Université Laval<br>Perreault , M; Douglas Mental Health University Institute,<br>Menear, Matthew; Universite Laval, Family Medicine and Emergency Medicine<br>Archambault, Leonie; Institut universitaire en santé mentale Douglas<br>Haagsma, Juanita; Erasmus MC, Public Health<br>Provencher, Helene; Université Laval<br>Genest, Christine; Université de Montréal, Faculté des sciences infirmières; Centre de recherche de l'Institut universitaire en sante mentale de Montreal,<br>Gagnon, Marc-Aurèle; Centre de recherche du CHU de Québec-Université Laval, Population Health and Optimal Practices<br>Bourque, Laurence; CHU de Quebec-Universite Laval, Population Health and Optimal Practices Research Unit Research Unit<br>Lapierre, Alexandra; Hôpital du Sacré-Coeur de Montréal Centre de Recherche,<br>Amal, Kalfi; Université Laval<br>Panenka, William; The University of British Columbia
**Primary Subject Heading**:	Mental health
Secondary Subject Heading:	Evidence based practice
Keywords:	TRAUMA MANAGEMENT, MENTAL HEALTH, Protocols & guidelines < HEALTH SERVICES ADMINISTRATION & MANAGEMENT, Systematic Review

For peer review only - http://bmjopen.bmj.com/site/about/guidelines.xhtml

Clinical practice guideline recommendations to improve the mental health of adult trauma patients: protocol for a systematic review

Mélanie Bérubé[1,2], Nori Bradley[3,4], Meaghan O'Donnell[5], Henry Thomas Stelfox[6], Naisan Garraway[7], Helen Maria Vasiliadis[8], Valérie Turcotte[2,9], Michel Perreault[10,11], Matthew Menear[12,13], Léonie Archambault[11], Juanita Haagsma[14], Hélène Provencher[2], Christine Genest[15], Marc-Aurèle Gagnon[1], Laurence Bourque[1], Alexandra Lapierre[1], Amal Khalfi[1,2], William Panenka[16,17]

[1]CHU de Québec-Université Laval Research Centre, Population Health and Optimal Practices Research Unit Research Unit (Trauma – Emergency – Critical Care Medicine), 1401, 18e rue, Québec City, Quebec, Canada, GIV 1Z4

[2]Faculty of Nursing, 1050 Avenue de la Médecine, Université Laval, Québec City, Quebec, Canada, G1V 0A6

[3]University of Albert Hospital, 8440 112 St NW, Edmonton, Alberta, Canada, T6G 2b7

[4]Royal Columbian Hospital, 330E Columbia St, New Westminster, British Columbia, V3L 3W7

[5]University of Melbourne, Department of Psychiatry, 161 Barry Street, Carlton VIC 30150

[6]University of Calgary & Alberta Health Services, O'Brien Institute for Public Health, 3280 Hospital Dr NW, Calgary, Alberta, Canada, T2N 4Z6

[7]Vancouver General Hospital, Trauma Services, 855 West 12 Avenue, Vancouver, British Columbia, Canada, V5Z 1M9

[8]Université de Sherbrooke, Community Health Sciences, 150 Place Charles Lemoyne, Longueil, Quebec, Canada, J4K 0A8

[9]CIUSSS du Nord-de-l'île-de-Montréal, Department of Nursing, 5400 Boulevard Gouin Ouest Montréal, Quebec, Canada, H4J 1C5

[10]McGill University, Department of Psychiatry, 1033 Pine Ave W, Montreal, Quebec, Canada, H3A 1A1

[11]Institut universitaire en santé mentale Douglas, 6875 Bd LaSalle, Verdun, Quebec, Canada, H4H 1R3

[12]Université Laval, Department of Family and Emergency Medicine, 1050 Avenue de la Médecine, Université Laval, Québec City, Quebec, Canada, G1V 0A6

[13]Centre de recherche en santé durable, Centre intégré universitaire de santé et de services sociaux de la Capitale-Nationale, 2480, chemin de la Canardière, Québec, Quebec, Canada, G1J 2G1

[14]Erasmus University, Department of Public Health, Dr. Molewaterplein 40, 3015 GD Rotterdam, Netherlands.

[15]Université de Montréal, Faculty of Nursing, 2375, chemin de la Côte-Ste-Catherine, Montreal, Quebec, Canada, H3T 1A8

[16]University of British Columbia, Department of Psychiatry, 2255 Wesbrook Mall, Vancouver, British Columbia, Canada, V6T 2A1

[17]British Columbia Mental health and Substance Use Services Research Institute, 938 West 28th Avenue, Vancouver, British Columbia, Canada, V5Z 4H4

Corresponding author:
Mélanie Bérubé
CHU de Québec-Université Laval Research Centre
1401, 18e rue, Québec City, Quebec, Canada, GIV 1Z4
T 418 649-0252 ext: 66600
email: melanie.berube@fsi.ulaval.ca

For peer review only

Abstract

Introduction: Mental disorders are common in adult patients with traumatic injuries. To limit the burden of poor psychological well-being in this population, recognized authorities have issued recommendations through clinical practice guidelines (CPGs). However, the uptake of evidence-based recommendations to improve the mental health of trauma patients has been low until recently. This may be explained by the complexity of optimizing mental health practices and interpreting CGPs scope and quality. Our aim is to systematically review CPG mental health recommendations in the context of trauma care and appraise their quality.

Methods and analysis: We will identify CPG through a search strategy applied to Medline, Embase, CINAHL, PsycINFO and Web of Science databases, as well as guidelines repositories and websites of trauma associations. We will target CPGs on adult and acute trauma populations including at least one recommendation on any prevention, screening, assessment, intervention, patient and family engagement, referral, or follow-up procedure related to mental health endorsed by recognized organizations in high-income countries. No language limitations will be applied, and we will limit the search to the last 15 years. Pairs of reviewers will independently screen titles, abstracts, full texts, and carry out data extraction and quality assessment of CPGs using the Appraisal of Guidelines Research and Evaluation (AGREE) II. We will synthesize the evidence on recommendations for CPGs rated as moderate or high quality using a matrix based on the GRADE quality of evidence, strength of recommendation, health and social determinants, and whether recommendations were made using a population-based approach.

Ethics and dissemination: Ethics approval is not required, as we will conduct secondary analysis of published data. The results will be disseminated in a peer-reviewed journal, at international and national scientific meetings. Accessible summary will be distributed to interested parties through professional, healthcare quality and persons with lived experience associations.

PROSPERO registration number : International Prospective Register of Systematic Reviews (ID454728)

Keywords: trauma care, mental health, best practices, implementation science

Strengths and limitations of this study

- We will produce a meta-synthesis of clinical practice guidelines (CPGs) recommendations on mental health in injury care based on their methodological quality and level of evidence.
- Our review involves interested parties to maximize its relevance and reach.
- We will focus solely on CPGs for mental health from high-income countries.
- Our search strategy is not designed to identify CPGs that do not specifically focus on mental health in trauma populations.

For peer review only - http://bmjopen.bmj.com/site/about/guidelines.xhtml

Introduction

Improvements in trauma care in high-income countries over the past 30 years have reduced mortality from as high as 50% to 11%.(1, 2) As a result, more and more patients survive their traumatic injuries, but still face a significant burden of morbidity. Indeed, traumatic injuries are a leading cause of disability (3,4), and result in the highest number of productive years of life lost.(5, 6)

The impact of traumatic injuries is not without consequences for patients' mental health, not to mention the fact that many are already vulnerable on a psychosocial level. Before the injury, 20-40% of trauma patients have mental health issues, including substance use disorders and mood disorders (7, 8), which can impair recovery.(9) After the injury, up to 45% of patients develop acute stress disorder during their hospital stay (10), and 10-50% post-traumatic stress disorder (PTSD) in many high-income countries, with prevalence varying according to trauma mechanisms and recovery phases.(9, 11-19) Hence, patients who sustained traumatic injuries have a 40% higher risk of being hospitalized for a mental disorder when comparing the five years before and after the injury.(19) Cumulative structural, social, and health determinants have been shown to contribute significantly to mental disorders among trauma patients with racial/ethnic minorities, those with low levels of education and financial resources, males or patients identifying themselves as men, and those living in rural areas being most vulnerable.(19, 20)

The high prevalence of mental disorders in trauma patients is alarming considering that they have profound negative impacts on individual and social outcomes. Trauma patients suffering from mental disorders were shown to have twice as many complications and twice the length of hospital stays.(21) Similarly, these patients are almost 10 times more likely to develop chronic pain and have physical limitations, and two to four times more likely not to return to work than patients without mental disorders (22), while having a poorer quality of life.(14)

For peer review only - http://bmjopen.bmj.com/site/about/guidelines.xhtml

Given the growing body of research evidence highlighting the burden of mental health issues following traumatic injury, recognized authorities (23-26) have proposed clinical practice guidelines (CPGs) to reduce their prevalence. CPG recommendations apply to all healthcare professionals (HCPs) involved in the trauma care continuum, and cover mental health promotion/illness prevention, screening, brief and more comprehensive interventions, patient referral to subsequent specialty services and follow-up, and patient and family centered-care approach [i.e., collaborative approach, share-decision making (27)]. HCPs' adherence rates to CPGs just prior to the publication of key CPGs were issued in late 2022, such as the American College of Surgeons-Committee on Trauma on Screening and Intervention for Substance Use in the Acute Trauma Patients (23), were are reported to be less than 30%.(28, 29) While uptake rates may have risen since then, the fact remains that improving mental health practices in an area where healthcare professionals have more expertise in physical health (19, 30) can be complex and put pressure on increasingly limited resources of healthcare systems. It is therefore crucial to summarize the recommendations on the prevention and treatment of mental disorders in the context of trauma, and identified those with the greatest support for implementation, to enable organizations in different countries to target the priorities on which to focus. Hence, we aim to systematically review CPG recommendations for mental health in the context of injury care, and to appraise their quality.

Methods

In line with the knowledge synthesis phase of the Knowledge to Action framework (31), we will conduct a knowledge synthesis on CPGs. This protocol was developed according to Cochrane recommendations for systematic reviews (SR) (32) and methodological guidelines for systematic review on CPGs (33) and is reported according to the Preferred Reporting Items for Systematic Review and Meta-Analysis Protocol statement.(34)

Patient and public involvement

To maximize contributions, we set up an advisory committee including representatives of associations that play a role in setting standards for trauma and mental health care (Trauma Association of Canada,

For peer review only - http://bmjopen.bmj.com/site/about/guidelines.xhtml

the Mental Health Commission of Canada, and the *Institut national d'excellence en santé et en services sociaux*) and in supporting persons with lived experience (Brain Injury Canada, Mothers Against Drunk Driving - Canada, *Moelle épinière et motricité Québec, and Connexion TCC-Québec*). The advisory committee also includes HCPs from various disciplines involved with trauma patients (specialized physicians/surgeons, nurses, rehabilitation professionals, psychologists, social workers), persons with lived experience, and decision makers. The committee was involved in the development of the protocol and will oversee the progress of the review and take part in disseminating the results.

Eligibility

We will target CPGs on adult (≥ 18 years old) and in-patient acute trauma populations (P) including at least one recommendation (R) on any prevention, screening, assessment, intervention, patient and family engagement, referral, or follow-up procedure related to mental health (I) with any or no comparator (C) endorsed by recognized organizations in high-income countries within the last 15 years (September 2008 to a maximum of six months prior to submission) (A). No language restriction will be applied. We will use an open-access online translator (https://www.deepl.com/translator) for studies not written in English of French.(35) CGPs are defined as "statements that include recommendations intended to optimize patient care that are informed by a review of evidence and an assessment of benefits and harms of alternative care options".(36) We will include CPGs focusing on the categories of mental disorders defined in the Diagnostic and Statistical Manual of Mental Disorders fifth edition-text revision (DSM-5-TR) (37) that most often affect trauma patients: trauma-and-stressor-related disorders, anxiety disorders, substance-related and addictive disorders, suicidal behavior disorder and self-injury.(7, 8, 19) High-income countries, based on World Bank definitions (38), will be targeted to ensure that recommendations are compatible with the practices of accredited trauma centers (39), while the time limit aims to focus on recent recommendations.

For peer review only - http://bmjopen.bmj.com/site/about/guidelines.xhtml

Search strategy

Based on the recommendations of the Peer Review of Electronic Search Strategies (40), we will establish a systematic search strategy in collaboration with an information specialist, which will be independently reviewed by a second information specialist. We will use a combination of controlled terms (e.g., MeSH for MEDLINE, Emtree for Embase) and free vocabulary according to the following themes: wounds and injuries, trauma, mental health, mental disorders, guidelines. We will apply the pilot search strategy in Medline (Ovid) and Embase (Ovid) databases. Relevant words found in the title, abstract and text of retrieved studies will be collected. We will then add these words and indexing terms to the search strategy, which will be executed in the Medline (Ovid), Embase (Ovid), CINAHL (EBSCO), PsycINFO (Ovid) and Web of Science (Clarivate) databases. Relevant GPGs will also be searched in CPGs repositories and trauma association websites, a list of which was drawn up with members of the research team who have in-depth knowledge of the innerworkings in this field. Using a preliminary search strategy (from January 2018 to August 8th 2023; table 1 and table 2), we have identified 3882 citations, including 5 sentinel CPGs (24, 25, 26, 41, 42) identified *a priori*, indicating good sensitivity and specificity.

CPGs selection and data extraction

We will manage citations using EndNote (version X9.3.3, New York City: Thomson Reuters, 2018). Pairs of reviewers will independently screen titles, abstracts and full-texts, and carry out data extraction. Once the final set of included CPGs has been obtained, all associated methodological supplements will be retrieved before data extraction or quality assessment begins.(33) Data from included CPGs will be extracted with an extraction form, which will be pilot-tested on five items to ensure feasibility and completeness.(32) For each recommendation within CPGs, we will extract information on the characteristics of included CPGs: title and year, country, organization, target users, population including determinants contributing to disparities in mental health care (43, 44) (e.g., type of trauma, gender, age group, ethnocultural group, geographical location, socioeconomic status), areas of focus, intervention, quality of evidence and strength of recommendations. Since interventions that use a population-based

For peer review only - http://bmjopen.bmj.com/site/about/guidelines.xhtml

approach, i.e. stepped and collaborative care tailored to patients' risks and needs, have been shown to be more effective than standardized clinical interventions in improving the mental health of trauma patients, we will also extract data on whether recommendations provide details on these aspects. If important information is missing or unclear, we will request it by sending up to three emails to the first, second and last authors.

Quality

Pairs of reviewers with content expertise will independently assess CPGs quality based on the six domains of the AGREE II tool (45): 1) scope and purpose (overall aim of the guideline; specific health questions; and target population), 2) interested parties involvement (developed by the appropriate stakeholders; consistent with the views of its intended users), 3) rigorously developed (process used to gather and synthesize the evidence; methods to formulate and update the recommendations), 4) clarity and presentation (language; structure; and format), 5) applicability (barriers and facilitators to implementation; strategies to improve uptake such as integration into a national trauma performance verification process, tools for use at an organizational level, billing guidance for mental health practices, links to other relevant guidelines and tools; demand on healthcare resources), and 6) editorial independence (competing interests reported and addressed). In accordance with guidelines on the systematic review of CPGs (33) and based on previous systematic reviews of CPGs (46-48), each domain with a score ≥60% will be considered effectively addressed. CPGs will be considered *high quality* if they score ≥60% in at least three of the six AGREE II domains, including domain 3 (rigor of development). If three domains or more score ≥60%, and domain 3 score <60%, the CPG will be considered *moderate quality*. CPGs scoring <60% in two domains or more and scoring <50% in domain 3 will be considered *low quality*.

Data synthesis

CPG recommendations will be classified into the following key areas, as determined by the advisory committee: 1) mental health promotion/illness prevention, 2) screening for mental disorders, 3)

For peer review only - http://bmjopen.bmj.com/site/about/guidelines.xhtml

assessment, 4) interventions, 5) referral for patients experiencing mental disorders for follow-up and support services, and 6) patient and family centered-care approach. We will synthesize the evidence on recommendations for CPGs rated as moderate or high quality (AGREE II) (45) using a recommendation matrix based on the GRADE quality of evidence (high, moderate, low and very low) (49), recommendation strength (low, high) (46-48), health and social determinants (i.e., care equity), and whether or not recommendations were formulated using a population-based approach. Matrix data will be extracted independently by pairs or reviewers for each recommendation. We will group the same or very similar recommendations published by more than one CPG. For CPGs that used a rating system other than GRADE, we will match reported categories to GRADE categories.

For selection, extraction, quality assessment and meta-synthesis, all reviewers will systematically and iteratively pilot test the process on a random selection of articles (200 citations for selection and 5 CPGs for extraction and quality evaluation and meta-synthesis in each iteration) until acceptable agreement is achieved (kappa>0.8).(32) Any discrepancies during the review process will be resolved by first consulting a senior member of the research team (WP), and then by reaching a consensus among the members of the advisory committee, if necessary.

Limitation of study

For feasibility reasons, our search strategy was not developed to systematically identify CPGs that do not specifically target mental disorders in trauma populations. Therefore, we may miss recommendations on mental health if they are included in CPGs that target trauma populations in general if no keywords relating to mental health and injury are present in the title or abstract. However, these recommendations are likely to be identified by consulting CPGs repositories and trauma association websites (Table 2), which were recognized as the best sources to find relevant guidelines.(50)

For peer review only - http://bmjopen.bmj.com/site/about/guidelines.xhtml

Potential impact

Recent data on mental health issues in trauma populations are now prompting recognized authorities (23-26) to prioritize this area to further improve the outcomes in injured patients. However, to date, there has been no synthesis of recommendations for improving mental health practices in the trauma field. This systematic review of CPGs will allow to identify recommendations with the greatest support for implementation, thus taking into account the staff shortage and time constraints omnipresent in healthcare systems. Results may be used by healthcare professionals, trauma program leaders, hospital administrators and policymakers to inform local quality improvement initiatives, identify system-wide problems and plan future actions to improve mental health practices.

Ethics and dissemination

Research ethics approval is not required, as we will conduct secondary analysis of published data. The results of our study will be disseminated in a peer-reviewed journal, at international and national scientific meetings, and an accessible summary will be distributed to stakeholders through professional, healthcare quality and persons with lived experience associations.

Author's contributions:

MB and WP led the development of the protocol and drafted the manuscript. They also secured funding and acts as guarantor for the review.

NB, MO, HTS drafted parts of the research methods, validated the search strategy, and critically appraised and approved the final manuscript.

NG, HMV assisted with the formulation of the background, objectives and inclusion criteria, and critically appraised and approved the final manuscript.

VT, MP, MM, LA, JH, HP, CG, AL, AK contributed to the development of research objectives and concept definitions, validated the search strategy, critically revised and approved the final version of the manuscript.

For peer review only - http://bmjopen.bmj.com/site/about/guidelines.xhtml

MAG and LB contributed to the formulation of research objectives and keywords, developed and tested the search strategy, and revised and approved the final version of the manuscript.

MO, MP, LA, HP, CG, WP acted as an expert in mental health and mental disorders and provided advice on how to integrate the concepts in the protocol.

Twitter:

@BrubMlanie1, @BradleyNori, @Turcottetrauma, @Alexe_Lapierre

Funding:

This work is supported by the Canadian Institutes for Health Research (#505680), Fonds de Recherche en Santé du Québec and the Strategy for Patient-Oriented Research-Quebec and the Quebec Nursing Intervention Network. Funders had not role in developing the protocol.

Competing interests:

None to declare

Patient and public involvement:

Patients with lived experience and public associations that play a role in setting standards for trauma and mental health care and in supporting persons with lived experience were involved in the design of this protocol and will be involved in the conduct, reporting, and dissemination of results associated with the proposed systematic review.

Provenance and peer reviewed:

Not commissioned; externally peer reviewed.

For peer review only

References

1. Moore L, Evans D, Hameed SM, *et al*. Mortality in Canadian Trauma Systems: A Multicenter Cohort Study. Ann Surg 2017; **265**(1): 212-17. doi: 10.1097/sla.0000000000001614
2. Truchon C, Moore L, Belcaid A, *et al*. Shaping Quality Through Vision, Structure, and Monitoring of Performance and Quality Indicators: Impact Story From the Quebec Trauma Network. Int J Technol Assess Health Care 2017;33(4):415-9. doi: 10.1017/s0266462317000198
3. World Health Organization. WHO reveals leading causes of death and disability worldwide: 2000-2019, 2020. Available: https://www.who.int/news/item/09-12-2020-who-reveals-leading-causes-of-death-and-disability-worldwide-2000-2019
4. GBD 2019 Diseases and Injuries Collaborators. Global burden of 369 diseases and injuries in 204 countries and territories, 1990–2019: a systematic analysis for the Global Burden of Disease Study 2019. Lancet 2020;396:1204-22. doi: 10.1016/S0140-6736(20)30925-9
5. Parachute. Cost of Injury in Canada, 2022. Available: https://parachute.ca/en/professional-resource/cost-of-injury-in-canada/
6. Centers for Disease Control and Prevention. Injury Prevention & Control: Cost of Injury Data, 2021. Available: https://www.cdc.gov/injury/wisqars/cost/
7. Clous EA, Beerthuizen KC, Ponsen KJ, *et al*. Trauma and psychiatric disorders: A systematic review. J Trauma Acute Care Surg 2017;82(4):794-801. doi: 10.1097/TA.0000000000001371.
8. Weinberg DS, Narayanan AS, Boden KA, *et al*. Psychiatric Illness Is Common Among Patients with Orthopaedic Polytrauma and Is Linked with Poor Outcomes. J Bone Joint Surg Am 2016;98(5):341-8. doi: 10.2106/JBJS.15.00751
9. Kruithof N, Polinder S, de Munter L, *et al*. Health status and psychological outcomes after trauma: A prospective multicenter cohort study. PLoS One 2020;15(4):e0231649. doi: 10.1371/journal.pone.0231649. eCollection 2020.
10. Wiseman T, Foster K, Curtis K. Mental health following traumatic physical injury: an integrative literature review. Injury 2013; 44(11):1383-90. doi: 10.1016/j.injury.2012.02.015. Epub 2012 Mar 10.
11. Lin W, Gong L, Xia M, Dai W. Prevalence of posttraumatic stress disorder among road traffic accident survivors: A PRISMA-compliant meta-analysis. Medicine (Baltimore) 2018;97(3):e9693. doi: 10.1097/MD.0000000000009693.
12. Roden-Foreman K, Solis J, Jones A, *et al*. Prospective Evaluation of Posttraumatic Stress Disorder and Depression in Orthopaedic Injury Patients With and Without Concomitant Traumatic Brain Injury. J Orthop Trauma 2017;**31**(9):e275-e80. doi: 10.1097/BOT.0000000000000884.
13. Joseph NM, Benedick A, Flanagan CD, *et al*. Risk Factors for Posttraumatic Stress Disorder in Acute Trauma Patients. J Orthop Trauma 2021;35(6):e209-e15. doi: 10.1097/BOT.0000000000001990.
14. van der Vlegel M, Polinder S, Toet H, *et al*. Anxiety, depression and post-traumatic stress symptoms among injury patients and the association with outcome after injury. Eur J Psychotraumatol 2022;13(1):2023422. doi: 10.1080/20008198.2021.2023422. eCollection 2022.
15. Warren AM, Jones AL, Bennett M, *et al*. Prospective Evaluation of Posttraumatic Stress Disorder in Injured Patients With and Without Orthopaedic Injury. J Orthop Trauma 2016;30(9):e305-11. doi: 10.1097/BOT.0000000000000623.
16. Bryant RA, O'Donnell ML, Creamer M, *et al*. The psychiatric sequelae of traumatic injury. Am J Psychiatry 2010;167(3):312-20. doi: 10.1176/appi.ajp.2009.09050617.

For peer review only - http://bmjopen.bmj.com/site/about/guidelines.xhtml

17. Zatzick DF, Rivara FP, Nathens, AB, *et al*. A nationwide study of post-traumatic stress after hospitalization for physical injury 2007;37(10):1469-80. https://doi.org/10.1017/S0033291707000943
18. Herrera-Escobar JP, Al Rafai SS, Seshadri AJ, *et al*. A multicenter study of post-traumatic stress disorder after injury: Mechanism matters more than injury severity. Surgery 2018;164(6):1246-50. doi: 10.1016/j.surg.2018.07.017. Epub 2018 Aug 29.
19. Evans CCD, DeWit Y, Seitz D, *et al*. Mental health outcomes after major trauma in Ontario: a population-based analysis. CMAJ 2018;190(45):E1319-e27. doi: 10.1503/cmaj.180368.
20 Mollayeva T, D'Souza A, Mollayeva S. Systematic Review of Sex and Gender Effects in Traumatic Brain Injury: Equity in Clinical and Functional Outcomes. Front Neurol 2021;12:678971. doi: 10.3389/fneur.2021.678971. eCollection 2021
21. Sharwood LN, Wiseman T, Tseris E, *et al*. Pre-existing mental disorder, clinical profile, inpatient services and costs in people hospitalised following traumatic spinal injury: a whole population record linkage study. Inj Prev 2020. doi: 10.1136/injuryprev-2019-043567
22. Herrera-Escobar JP, Seshadri AJ, Stanek E, *et al*. Mental Health Burden After Injury: It's About More than Just Posttraumatic Stress Disorder. Ann Surg 2021;274(6):e1162-e9. doi: 10.1097/SLA.0000000000003780
23. Committee on Trauma-American College of Surgeons. Best Practices Guidelines: Screening and Interventions for Mental Health Disorders and Subctance Use in the Acute Trauma Patients, 2022. Available: https://www.facs.org/quality-programs/trauma/quality/best-practices-guidelines/
24. Substance Abuse and Mental Health Services Administration. Substance Use Disorder Treatment for People With Physical and Cognitive Disabilities Advisory, 2019. Available: https://store.samhsa.gov/sites/default/files/d7/priv/pep19-02-00002_508_022620.pdf
25. Bombardier CH, Azuero CB, Fann JR, *et al*. Management of Mental Health Disorders, Substance Use Disorders, and Suicide in Adults with Spinal Cord Injury: Clinical Practice Guideline for Healthcare Providers. Top Spinal Cord Inj Rehabil 2021;27(2):152-224. doi: 10.46292/sci2702-152
26. Major Extremity trauma and Rehabilitation Consortium and the American Academy of Orthopaedic Surgeons. Evaluation of Psychosocial Factors Influencing Recovery from Adult Orthopeadic Trauma, 2019. Available: https://www.aaos.org/globalassets/quality-and-practice-resources/dod/prf-cpg-final-draft-12-3-19.pdf
27. World Health Organization. WHO global strategy on people-centred and integrated health services, 2015. Available: https://interprofessional.global/wp-content/uploads/2019/11/WHO-2015-Global-strategy-on-integrated-people-centred-health-services-2016-2026.pdf
28. Lamontagne ME, Gargaro J, Marier-Deschênes P, *et al.* A Survey of Perceived Implementation Gaps for a Clinical Practice Guideline for the Rehabilitation of Adults With Moderate to Severe Traumatic Brain Injury. J Head Trauma Rehabil 2018;33(5):306-16. doi: 10.1097/HTR.0000000000000430
29. Bulger EM, Johnson P, Parker L, *et al*. Nationwide Survey of Trauma Center Screening and Intervention Practices for Posttraumatic Stress Disorder, Firearm Violence, Mental Health, and Substance Use Disorders. J Am Coll Surg 2022;234(3):274-87. doi: 10.1097/XCS.0000000000000064
30. Sinkler MA, Furdock RJ, Vallier HA. Treating trauma more effectively: A review of psychosocial programming. Injury. 2022;53(6):1756-64. doi: 10.1016/j.injury.2022.04.02
31. Graham ID, Logan J, Harrison MB, *et al*. Lost in knowledge translation: time for a map? J Contin Educ Health Prof 2006;26(1):13-24. doi: 10.1002/chp.47
32. Higgins J TJ, Chandler J, Cumpston M, Li T, *et al*. Cochrane Handbook for Systematic Reviews of Interventions version 6.3, 2022. Available: www.training.cochrane.org/handbook

For peer review only - http://bmjopen.bmj.com/site/about/guidelines.xhtml

33. Johnston A, Kelly SE, Hsieh SC, *et al*. Systematic reviews of clinical practice guidelines: a methodological guide. J Clin Epidemiol 2019;108:64-76. doi: 10.1016/j.jclinepi.2018.11.030. Epub 2018 Dec 5.
34. Moher D, Shamseer L, Clarke M, *et al*. Preferred reporting items for systematic review and meta-analysis protocols (PRISMA-P) 2015 statement. Systematic Reviews 2015;4(1):1. doi: 10.1186/2046-4053-4-1
35. Takakusagi Y, Oike T, Shirai K, *et al*. Validation of the Reliability of Machine Translation for a Medical Article From Japanese to English Using DeepL Translator. Cureus 2021; 13(9): e17778. doi: 10.7759/cureus.17778. eCollection 2021 Sep.
36. Institute of Medicine (US) Committee on Standards for Developing Trustworthy Clinical Practice Guidelines, *Clinical Practice Guidelines We Can Trust*, ed. R. Graham, M. Mancher, and W.D. Miller. 2011: Washington (DC): National Academies Press (US).
37. American Psychiatric Association. Diagnostic and Statistical Manual of Mental Disorders. Fifth Edition, Text-Revision (DSM-TR), 2022: Washington (DC), US.
38. Blogs WB. New World Bank country classifications by income level: 2022-2023. [cited 2023 August 20th]. Available: https://blogs.worldbank.org/opendata/new-world-bank-country-classifications-income-level-2022-2023
39. Committee on Trauma-American College of Surgeons. Resources for Optimal Care of the Injured Patient, 2022. Available: https://www.facs.org/quality-programs/trauma/quality/verification-review-and-consultation-program/standards/
40. McGowan J, Sampson M, Salzwedel DM, *et al*. PRESS Peer Review of Electronic Search Strategies: 2015 Guideline Statement. J Clin Epidemiol 2016;75:40-6. doi: 10.1016/j.jclinepi.2016.01.021
41. Metha S OS, Blackport D, Teasell R. Mental Health after a Spinal Cord Injury, 2022. Available: https://scireproject.com/evidence/mental-health/introduction/
42. Kodadek LM, Freeman JJ, Tiwary D, *et al*. Alcohol-related trauma reinjury prevention with hospital-based screening in adult populations: An Eastern Association for the Surgery of Trauma evidence-based systematic review. J Trauma Acute Care Surg 2020;88(1):106-12. doi: 10.1097/ta.0000000000002501
43. Haider AH, Weygandt PL, Bentley JM, *et al*. Disparities in trauma care and outcomes in the. United States: a. systematic review and meta-analysis. J. Trauma Acute care Surg 2013;74(5);1195-205. doi: 10.1097/TA.0b013e31828c331d
44. Englum BR, Villegas C, Bolorunduro O, *et al*. Racial, ethnic, and insurance status disparities in use of posthospitalization care after trauma. J Am Coll Surg 2011;213(6):699-708. doi: 10.1016/j.jamcollsurg.2011.08.017
45. Brouwers MC, Kho ME, Browman GP, *et al*. AGREE II: advancing guideline development, reporting and evaluation in health care. Cmaj 2010;182(18):E839-42. doi: 10.1503/cmaj.090449
46. Ben Abdeljelil A, Freire GC, Yanchar N, *et al*. Pediatric Moderate and Severe Traumatic Brain Injury: A Systematic Review of Clinical Practice Guideline Recommendations. J Neurotrauma 2023 (ahead of print). doi: 10.1089/neu.2023.0149
47. Freire GC, Beno S, Yanchar N, *et al*. Clinical Practice Guideline Recommendations For Pediatric Multisystem Trauma Care: A Systematic Review. Ann Surg 2023 (ahead of print). doi: 10.1097/SLA.0000000000005966
48. Yanchar N, Tardif PA, Freire G, e*t al*. Clinical Practice Guidelines Recommendations for Pediatric Solid Organ Injury Care: A Systematic Review. J Trauma Acute Care Surg 2023 (ahead of print). doi: 10.1097/TA.0000000000004015
49. Moberg J, Oxman AD, Rosenbaum S, *et al*. The GRADE Evidence to Decision (EtD) framework for health system and public health decisions. Health Res Policy Syst 2018;16(1):45. doi: 10.1186/s12961-018-0320-2

For peer review only

50. Lunny C, Salzwedel DM, Liu T, *et al*. Validation of five search filters for retrieval of clinical practice guidelines produced low precision. J Clin Epidemiol 2020;117:109-16. doi: 10.1016/j.jclinepi.2019.09.022

For peer review only

Table 1. Medline (Ovid) (August 8th, 2023)

Concepts	Search strategy keywords	Research and no of results
**Trauma/ recovery** (Controlled vocabulary)	exp "Wounds and Injuries"/ or exp Brain Hemorrhage, Traumatic/ or Brain Injuries/ or Coma, Post-Head Injury/ or Craniocerebral Trauma/ or Diffuse Axonal Injury/ or exp Fractures, Bone/ or Head Injuries, Closed/ or Head Injuries, Penetrating/ or exp Intracranial Hemorrhage, Traumatic/ or exp Skull Fractures/	#1<br>1 007 620
	Fractur* OR Injur* OR TBI OR trauma*	#2<br>1 430 550
(Free text)	1 OR 2	#3<br>1 894 323
**Mental health** (Controlled vocabulary)	exp Mental Disorders/ or exp Mental Health/ or exp Substance-Related Disorders/	#4<br>1 501 202
(Free text)	"mental health" OR "mental disorder*" OR "mental illness" OR "post-traumatic stress disorder" OR "post traumatic stress disorder" OR "PTSD" OR "Acute stress disorder*" or "acute stress reaction*" or "adjustment disorder*" or "suicidal behavior*" or "self-injur*" or "self injur*" or "self-harm" or "self harm" or "depressive disorder*" or "depression" or "mood disorder*" or "anxiety disorder*" or "trauma and stressor-related disorder*" or "trauma and stressor related disorder*" or "Mental distress" or "psychological distress" OR ((drug* OR substance OR alcohol OR marijuana OR cannabis OR narcotic* OR opiate* OR opioid* OR opium) Adj4 (abus* OR "use" OR user OR usage OR misus* OR usin* OR utilis* OR depend* OR addict* OR illegal* OR illicit* OR habit* OR withdraw* OR behavi* OR abstinence* OR abstain* OR intoxica* OR addict* OR disorder*)) OR "drug rehabilitation" OR "non-prescription drugs" OR "non-prescription drug"	#5<br>1 069 467
	4 OR 5	#6<br>2 121 781
**Guideline** (Controlled vocabulary)	exp Practice Guidelines as Topic/ or exp Practice Guideline/ or exp Guidelines as Topic/ or exp Guideline/	#7<br>208 566
(Free text)	Guide OR Guideline*	#8<br>676 237
	7 OR 8	#9<br>784 520
**Total**	3 AND 6 AND 9<br>Limit to 2008-2023	3 882

For peer review only

Table 2. Preliminary list of clinical practice guidelines repositories and trauma professional associations

1-Agency for Healthcare Research and Quality	32-Guideline Central
2-Accreditation Canada	33- Guidelines International Network
3- Australasian Association for Quality in Healthcare	34-Headway—the brain injury association
4-American Academy of Orthopedic Surgeons	35- International Association for Trauma Surgery and Intensive Care
5-American Association for the Surgery on Trauma	36-International Society of Orthopaedic and Traumatology
6- American Association of Neurological Surgeons	37-Italian Society of Orthopaedics and Traumatology
7- American Board of Orthopedic Surgery	38- National Association for Healthcare Quality
8-American College of Surgeons	39-National Guidelines Clearinghouse
9-American Orthopaedic Association	40- National Institute of Health and Care Excellence
10-American Spinal Injury Association	41- National Quality Forum
11-American Trauma Society	42-Norwegian Neurosurgical Association
12-Australian Society of Orthopeadic Surgeons	43-Ontario Neurotrauma Foundation
13- Australian and New Zealand Society	44- Orthopedic Trauma Association
14-Belgian Society for Orthopedics and Traumatology	45-Scandinavian Neurotrauma Committee
15-Brain Injury Association of America	46-Scottish Intercollegiate Guidelines Network
16-Brain Trauma Foundation	47-Société Française de Chirurgie Orthopédique et Traumatologique
17- British Orthopaedic Association	48-Society of Critical Care Medicine Trauma Network
18-British Trauma Society	49-Société Royale Belge de Chirurgie Orthopédique et de Traumatologie
19- Canada's Drug and Health Technology Agency	50-Spanish Society of Orthopaedic Surgery and Traumatology
20-Centers for Disease Control and Prevention	51- Society of Trauma Nurses
21-Congress of Neurological Surgeons	52-Spinal Cord Injury Research Evidence
22- Consortium of Spinal Cord Medicine	53-Sustance Abuse and Mental Health Services Administrations

For peer review only

23-Danish Neurosurgical Society	54-Swedish Neurosurgical Societ
24-Dutch Institute for Healthcare Improvement	55-Trauma Association of Canada
25- Eastern Association for the Surgery of Trauma	56-Trauma Audit Research Network
26-European Association of Neurosurgical Societies	57-Trauma.org
27-European Federations of National Associations of Orthopaedic and Traumatology	58-U.S. Department of Health and Human Services
28-European Society for Trauma and Emergency Surgery	59-U.S. Department of Veterans Affairs
29-Finnish Association of Neuroscience Nurses	60-Western Trauma Association
30-Finnish Neurosurgical Society	61-World Health Organization
31-Greek Society of Orthopaedic and Traumatological Surgery	

For peer review only

PRISMA-P (Preferred Reporting Items for Systematic review and Meta-Analysis Protocols) 2015 checklist: recommended items to address in a systematic review protocol*

Section and topic	Item No	Checklist item	Page
**ADMINISTRATIVE INFORMATION**			
Title:			
Identification	1a	Identify the report as a protocol of a systematic review	Page 1
Update	1b	If the protocol is for an update of a previous systematic review, identify as such	NA
Registration	2	If registered, provide the name of the registry (such as PROSPERO) and registration number	Page 2
Authors:			
Contact	3a	Provide name, institutional affiliation, e-mail address of all protocol authors; provide physical mailing address of corresponding author	Page 1
Contributions	3b	Describe contributions of protocol authors and identify the guarantor of the review	Page 9
Amendments	4	If the protocol represents an amendment of a previously completed or published protocol, identify as such and list changes; otherwise, state plan for documenting important protocol amendments	NA
Support:			
Sources	5a	Indicate sources of financial or other support for the review	Page 9
Sponsor	5b	Provide name for the review funder and/or sponsor	Page 9
Role of sponsor or funder	5c	Describe roles of funder(s), sponsor(s), and/or institution(s), if any, in developing the protocol	Page 9
**INTRODUCTION**			
Rationale	6	Describe the rationale for the review in the context of what is already known	Pages 3-4
Objectives	7	Provide an explicit statement of the question(s) the review will address with reference to participants, interventions, comparators, and outcomes (PICO)	Page 5
**METHODS**			
Eligibility criteria	8	Specify the study characteristics (such as PICO, study design, setting, time frame) and report characteristics (such as years considered, language, publication status) to be used as criteria for eligibility for the review	Pages 5-6
Information sources	9	Describe all intended information sources (such as electronic databases, contact with study authors, trial registers or other grey literature sources) with planned dates of coverage	Pages 5-6
Search strategy	10	Present draft of search strategy to be used for at least one electronic database, including planned limits, such that it could be repeated	Tables 1 and 2
Study records:			

For peer review only

Data management	11a	Describe the mechanism(s) that will be used to manage records and data throughout the review	Pages 6-7
Selection process	11b	State the process that will be used for selecting studies (such as two independent reviewers) through each phase of the review (that is, screening, eligibility and inclusion in meta-analysis)	Pages 6-7
Data collection process	11c	Describe planned method of extracting data from reports (such as piloting forms, done independently, in duplicate), any processes for obtaining and confirming data from investigators	Pages 6, 7, 8
Data items	12	List and define all variables for which data will be sought (such as PICO items, funding sources), any pre-planned data assumptions and simplifications	Pages 6-7
Outcomes and prioritization	13	List and define all outcomes for which data will be sought, including prioritization of main and additional outcomes, with rationale	Page 6
Risk of bias in individual studies	14	Describe anticipated methods for assessing risk of bias of individual studies, including whether this will be done at the outcome or study level, or both; state how this information will be used in data synthesis	Page 7
Data synthesis	15a	Describe criteria under which study data will be quantitatively synthesised	Pages 7-8
	15b	If data are appropriate for quantitative synthesis, describe planned summary measures, methods of handling data and methods of combining data from studies, including any planned exploration of consistency (such as I^2, Kendall's τ)	NA
	15c	Describe any proposed additional analyses (such as sensitivity or subgroup analyses, meta-regression)	Page 8
	15d	If quantitative synthesis is not appropriate, describe the type of summary planned	Page 8
Meta-bias(es)	16	Specify any planned assessment of meta-bias(es) (such as publication bias across studies, selective reporting within studies)	NA
Confidence in cumulative evidence	17	Describe how the strength of the body of evidence will be assessed (such as GRADE)	NA

*** It is strongly recommended that this checklist be read in conjunction with the PRISMA-P Explanation and Elaboration (cite when available) for important clarification on the items. Amendments to a review protocol should be tracked and dated.** **The copyright for PRISMA-P (including checklist) is held by the PRISMA-P Group and is distributed under a Creative Commons Attribution Licence 4.0.**

From: Shamseer L, Moher D, Clarke M, Ghersi D, Liberati A, Petticrew M, Shekelle P, Stewart L, PRISMA-P Group. Preferred reporting items for systematic review and meta-analysis protocols (PRISMA-P) 2015: elaboration and explanation. BMJ. 2015 Jan 2;349(jan02 1):g7647.

For peer review only

BMJ Open

Clinical practice guideline recommendations to improve the mental health of adult trauma patients: protocol for a systematic review

Journal:	*BMJ Open*
Manuscript ID	bmjopen-2023-079205.R2
Article Type:	Protocol
Date Submitted by the Author:	11-Mar-2024
Complete List of Authors:	Berube, Melanie; CHU de Québec-Université Laval Research Centre, Population Health and Optimal Practices Research Unit Research Unit (Trauma – Emergency – Critical Care Medicine); Universite Laval, Faculty of Nursing<br>Bradley, Nori; University of Alberta Hospital<br>O'Donnell, Meaghan; The University of Melbourne Faculty of Medicine Dentistry and Health Sciences, Department of Psychiatry<br>Stelfox, Henry; University of Calgary, Critical Care Medicine<br>Garraway, Naisan; Vancouver General Hospital<br>Vasiliadis, Helen-Maria; University of Sherbrooke<br>Turcotte, Valerie; Université Laval<br>Perreault , M; Institut universitaire en santé mentale Douglas<br>Menear, Matthew; Universite Laval, Family Medicine and Emergency Medicine<br>Archambault, Leonie; Institut universitaire en santé mentale Douglas<br>Haagsma, Juanita; Erasmus MC, Public Health<br>Provencher, Helene; Université Laval, Faculty of Nursing<br>Genest, Christine; Université de Montréal, Faculté des sciences infirmières; Centre de recherche de l'Institut universitaire en sante mentale de Montreal,<br>Gagnon, Marc-Aurèle; Centre de recherche du CHU de Québec-Université Laval, Population Health and Optimal Practices<br>Bourque, Laurence; CHU de Quebec-Universite Laval, Population Health and Optimal Practices Research Unit Research Unit<br>Lapierre, Alexandra; CHU de Québec-Université Laval Research Centre, Population Health and Optimal Practices Research Unit Research Unit (Trauma – Emergency – Critical Care Medicine)<br>Amal, Kalfi; Université Laval<br>Panenka, William; The University of British Columbia
**Primary Subject Heading**:	Mental health
Secondary Subject Heading:	Evidence based practice
Keywords:	TRAUMA MANAGEMENT, MENTAL HEALTH, Protocols & guidelines < HEALTH SERVICES ADMINISTRATION & MANAGEMENT, Systematic Review

SCHOLARONE™
Manuscripts

Clinical practice guideline recommendations to improve the mental health of adult trauma patients: protocol for a systematic review

Mélanie Bérubé[1,2], Nori Bradley[3,4], Meaghan O'Donnell[5], Henry Thomas Stelfox[6], Naisan Garraway[7], Helen Maria Vasiliadis[8], Valérie Turcotte[2,9], Michel Perreault[10,11], Matthew Menear[12,13], Léonie Archambault[11], Juanita Haagsma[14], Hélène Provencher[2], Christine Genest[15], Marc-Aurèle Gagnon[1], Laurence Bourque[1], Alexandra Lapierre[1], Amal Khalfi[1,2], William Panenka[16,17]

[1]CHU de Québec-Université Laval Research Centre, Population Health and Optimal Practices Research Unit Research Unit (Trauma – Emergency – Critical Care Medicine), 1401, 18e rue, Québec City, Quebec, Canada, GIV 1Z4

[2]Faculty of Nursing, 1050 Avenue de la Médecine, Université Laval, Québec City, Quebec, Canada, G1V 0A6

[3]University of Albert Hospital, 8440 112 St NW, Edmonton, Alberta, Canada, T6G 2b7

[4]Royal Columbian Hospital, 330E Columbia St, New Westminster, British Columbia, V3L 3W7

[5]University of Melbourne, Department of Psychiatry, 161 Barry Street, Carlton VIC 30150

[6]University of Calgary & Alberta Health Services, O'Brien Institute for Public Health, 3280 Hospital Dr NW, Calgary, Alberta, Canada, T2N 4Z6

[7]Vancouver General Hospital, Trauma Services, 855 West 12 Avenue, Vancouver, British Columbia, Canada, V5Z 1M9

[8]Université de Sherbrooke, Community Health Sciences, 150 Place Charles Lemoyne, Longueil, Quebec, Canada, J4K 0A8

[9]CIUSSS du Nord-de-l'île-de-Montréal, Department of Nursing, 5400 Boulevard Gouin Ouest Montréal, Quebec, Canada, H4J 1C5

[10]McGill University, Department of Psychiatry, 1033 Pine Ave W, Montreal, Quebec, Canada, H3A 1A1

[11]Institut universitaire en santé mentale Douglas, 6875 Bd LaSalle, Verdun, Quebec, Canada, H4H 1R3

[12]Université Laval, Department of Family and Emergency Medicine, 1050 Avenue de la Médecine, Université Laval, Québec City, Quebec, Canada, G1V 0A6

[13]Centre de recherche en santé durable, Centre intégré universitaire de santé et de services sociaux de la Capitale-Nationale, 2480, chemin de la Canardière, Québec, Quebec, Canada, G1J 2G1

[14]Erasmus University, Department of Public Health, Dr. Molewaterplein 40, 3015 GD Rotterdam, Netherlands.

[15]Université de Montréal, Faculty of Nursing, 2375, chemin de la Côte-Ste-Catherine, Montreal, Quebec, Canada, H3T 1A8

[16]University of British Columbia, Department of Psychiatry, 2255 Wesbrook Mall, Vancouver, British Columbia, Canada, V6T 2A1

[17]British Columbia Mental health and Substance Use Services Research Institute, 938 West 28th Avenue, Vancouver, British Columbia, Canada, V5Z 4H4

Corresponding author:
Mélanie Bérubé
CHU de Québec-Université Laval Research Centre
1401, 18e rue, Québec City, Quebec, Canada, GIV 1Z4
T 418 649-0252 ext: 66600
email: melanie.berube@fsi.ulaval.ca

For peer review only

Abstract

Introduction: Mental disorders are common in adult patients with traumatic injuries. To limit the burden of poor psychological well-being in this population, recognized authorities have issued recommendations through clinical practice guidelines (CPGs). However, the uptake of evidence-based recommendations to improve the mental health of trauma patients has been low until recently. This may be explained by the complexity of optimizing mental health practices and interpreting CGPs scope and quality. Our aim is to systematically review CPG mental health recommendations in the context of trauma care and appraise their quality.

Methods and analysis: We will identify CPG through a search strategy applied to Medline, Embase, CINAHL, PsycINFO and Web of Science databases, as well as guidelines repositories and websites of trauma associations. We will target CPGs on adult and acute trauma populations including at least one recommendation on any prevention, screening, assessment, intervention, patient and family engagement, referral, or follow-up procedure related to mental health endorsed by recognized organizations in high-income countries. No language limitations will be applied, and we will limit the search to the last 15 years. Pairs of reviewers will independently screen titles, abstracts, full texts, and carry out data extraction and quality assessment of CPGs using the Appraisal of Guidelines Research and Evaluation (AGREE) II. We will synthesize the evidence on recommendations for CPGs rated as moderate or high quality using a matrix based on the GRADE quality of evidence, strength of recommendation, health and social determinants, and whether recommendations were made using a population-based approach.

Ethics and dissemination: Ethics approval is not required, as we will conduct secondary analysis of published data. The results will be disseminated in a peer-reviewed journal, at international and national scientific meetings. Accessible summary will be distributed to interested parties through professional, healthcare quality and persons with lived experience associations.

PROSPERO registration number : International Prospective Register of Systematic Reviews (ID454728)

Keywords: trauma care, mental health, best practices, implementation science

Strengths and limitations of this study

- We will produce a meta-synthesis of clinical practice guidelines (CPGs) recommendations on mental health in injury care based on their methodological quality and level of evidence.
- Our review involves interested parties to maximize its relevance and reach.
- We will focus solely on CPGs for mental health from high-income countries.
- Our search strategy is not designed to identify CPGs that do not specifically focus on mental health in trauma populations.

For peer review only - http://bmjopen.bmj.com/site/about/guidelines.xhtml

Introduction

Improvements in trauma care in high-income countries over the past 30 years have reduced mortality from as high as 50% to 11%.(1, 2) As a result, more and more patients survive their traumatic injuries, but still face a significant burden of morbidity. Indeed, traumatic injuries are a leading cause of disability (3,4), and result in the highest number of productive years of life lost.(5, 6)

The impact of traumatic injuries is not without consequences for patients' mental health, not to mention the fact that many are already vulnerable on a psychosocial level. Before the injury, 20-40% of trauma patients have mental health issues, including substance use disorders and mood disorders (7, 8), which can impair recovery.(9) After the injury, up to 45% of patients develop acute stress disorder during their hospital stay (10), and 10-50% post-traumatic stress disorder (PTSD) in many high-income countries, with prevalence varying according to trauma mechanisms and recovery phases.(9, 11-19) Hence, patients who sustained traumatic injuries have a 40% higher risk of being hospitalized for a mental disorder when comparing the five years before and after the injury.(19) Cumulative structural, social, and health determinants have been shown to contribute significantly to mental disorders among trauma patients with racial/ethnic minorities, those with low levels of education and financial resources, males or patients identifying themselves as men, and those living in rural areas being most vulnerable.(19, 20)

The high prevalence of mental disorders in trauma patients is alarming considering that they have profound negative impacts on individual and social outcomes. Trauma patients suffering from mental disorders were shown to have twice as many complications and twice the length of hospital stays.(21) Similarly, these patients are almost 10 times more likely to develop chronic pain and have physical limitations, and two to four times more likely not to return to work than patients without mental disorders (22), while having a poorer quality of life.(14)

For peer review only

Given the growing body of research evidence highlighting the burden of mental health issues following traumatic injury, recognized authorities (23-26) have proposed clinical practice guidelines (CPGs) to reduce their prevalence. CPG recommendations apply to all healthcare professionals (HCPs) involved in the trauma care continuum, and cover mental health promotion/illness prevention, screening, brief and more comprehensive interventions, patient referral to subsequent specialty services and follow-up, and patient and family centered-care approach [i.e., collaborative approach, share-decision making (27)]. HCPs' adherence rates to CPGs just prior to the publication of key CPGs were issued in late 2022, such as the American College of Surgeons-Committee on Trauma on Screening and Intervention for Substance Use in the Acute Trauma Patients (23), were are reported to be less than 30%.(28, 29) While uptake rates may have risen since then, the fact remains that improving mental health practices in an area where healthcare professionals have more expertise in physical health (19, 30) can be complex and put pressure on increasingly limited resources of healthcare systems. It is therefore crucial to summarize the recommendations on the prevention and treatment of mental disorders in the context of trauma, and identified those with the greatest support for implementation, to enable organizations in different countries to target the priorities on which to focus. Hence, we aim to systematically review CPG recommendations for mental health in the context of injury care, and to appraise their quality.

Methods

In line with the knowledge synthesis phase of the Knowledge to Action framework (31), we will conduct a knowledge synthesis on CPGs. This protocol was developed according to Cochrane recommendations for systematic reviews (SR) (32) and methodological guidelines for systematic review on CPGs (33) and is reported according to the Preferred Reporting Items for Systematic Review and Meta-Analysis Protocol statement.(34)

Patient and public involvement

To maximize contributions, we set up an advisory committee including representatives of associations that play a role in setting standards for trauma and mental health care (Trauma Association of Canada,

For peer review only - http://bmjopen.bmj.com/site/about/guidelines.xhtml

the Mental Health Commission of Canada, and the *Institut national d'excellence en santé et en services sociaux*) and in supporting persons with lived experience (Brain Injury Canada, Mothers Against Drunk Driving - Canada, *Moelle épinière et motricité Québec, and Connexion TCC-Québec*). The advisory committee also includes HCPs from various disciplines involved with trauma patients (specialized physicians/surgeons, nurses, rehabilitation professionals, psychologists, social workers), persons with lived experience, and decision makers. The committee was involved in the development of the protocol and will oversee the progress of the review and take part in disseminating the results.

Eligibility

We will target CPGs on adult (≥ 18 years old) and in-patient acute trauma populations (P) including at least one recommendation (R) on any prevention, screening, assessment, intervention, patient and family engagement, referral, or follow-up procedure related to mental health (I) with any or no comparator (C) endorsed by recognized organizations in high-income countries within the last 15 years (September 2008 to a maximum of six months prior to submission) (A). No language restriction will be applied. We will use an open-access online translator (https://www.deepl.com/translator) for studies not written in English of French.(35) CGPs are defined as "statements that include recommendations intended to optimize patient care that are informed by a review of evidence and an assessment of benefits and harms of alternative care options".(36) We will include CPGs focusing on the categories of mental disorders defined in the Diagnostic and Statistical Manual of Mental Disorders fifth edition-text revision (DSM-5-TR) (37) that most often affect trauma patients: trauma-and-stressor-related disorders, anxiety disorders, substance-related and addictive disorders, suicidal behavior disorder and self-injury.(7, 8, 19) High-income countries, based on World Bank definitions (38), will be targeted to ensure that recommendations are compatible with the practices of accredited trauma centers (39), while the time limit aims to focus on recent recommendations.

For peer review only - http://bmjopen.bmj.com/site/about/guidelines.xhtml

Search strategy

Based on the recommendations of the Peer Review of Electronic Search Strategies (40), we will establish a systematic search strategy in collaboration with an information specialist, which will be independently reviewed by a second information specialist. We will use a combination of controlled terms (e.g., MeSH for MEDLINE, Emtree for Embase) and free vocabulary according to the following themes: wounds and injuries, trauma, mental health, mental disorders, guidelines. We will apply the pilot search strategy in Medline (Ovid) and Embase (Ovid) databases. Relevant words found in the title, abstract and text of retrieved studies will be collected. We will then add these words and indexing terms to the search strategy, which will be executed in the Medline (Ovid), Embase (Ovid), CINAHL (EBSCO), PsycINFO (Ovid) and Web of Science (Clarivate) databases. Relevant GPGs will also be searched in CPGs repositories and trauma association websites, a list of which was drawn up with members of the research team who have in-depth knowledge of the innerworkings in this field. Using a preliminary search strategy (from January 2018 to August 8th 2023; table 1 and table 2), we have identified 3882 citations, including 5 sentinel CPGs (24, 25, 26, 41, 42) identified *a priori*, indicating good sensitivity and specificity.

CPGs selection and data extraction

We will manage citations using EndNote (version X9.3.3, New York City: Thomson Reuters, 2018). Pairs of reviewers will independently screen titles, abstracts and full-texts, and carry out data extraction. Once the final set of included CPGs has been obtained, all associated methodological supplements will be retrieved before data extraction or quality assessment begins.(33) Data from included CPGs will be extracted with an extraction form, which will be pilot-tested on five items to ensure feasibility and completeness.(32) For each recommendation within CPGs, we will extract information on the characteristics of included CPGs: title and year, country, organization, target users, population including determinants contributing to disparities in mental health care (43, 44) (e.g., type of trauma, gender, age group, ethnocultural group, geographical location, socioeconomic status), areas of focus, intervention, quality of evidence and strength of recommendations. Since interventions that use a population-based

For peer review only - http://bmjopen.bmj.com/site/about/guidelines.xhtml

approach, i.e. stepped and collaborative care tailored to patients' risks and needs, have been shown to be more effective than standardized clinical interventions in improving the mental health of trauma patients (45), we will also extract data on whether recommendations provide details on these aspects. If important information is missing or unclear, we will request it by sending up to three emails to the first, second and last authors.

Quality

Pairs of reviewers with content expertise will independently assess CPGs quality based on the six domains of the AGREE II tool (46): 1) scope and purpose (overall aim of the guideline; specific health questions; and target population), 2) interested parties involvement (developed by the appropriate stakeholders; consistent with the views of its intended users), 3) rigorously developed (process used to gather and synthesize the evidence; methods to formulate and update the recommendations), 4) clarity and presentation (language; structure; and format), 5) applicability (barriers and facilitators to implementation; strategies to improve uptake such as integration into a national trauma performance verification process, tools for use at an organizational level, billing guidance for mental health practices, links to other relevant guidelines and tools; demand on healthcare resources), and 6) editorial independence (competing interests reported and addressed). In accordance with guidelines on the systematic review of CPGs (33) and based on previous systematic reviews of CPGs (47-49), each domain with a score ≥60% will be considered effectively addressed. CPGs will be considered *high quality* if they score ≥60% in at least three of the six AGREE II domains, including domain 3 (rigor of development). If three domains or more score ≥60%, and domain 3 score <60%, the CPG will be considered *moderate quality*. CPGs scoring <60% in two domains or more and scoring <50% in domain 3 will be considered *low quality*.

Data synthesis

CPG recommendations will be classified into the following key areas, as determined by the advisory committee: 1) mental health promotion/illness prevention, 2) screening for mental disorders, 3)

For peer review only - http://bmjopen.bmj.com/site/about/guidelines.xhtml

assessment, 4) interventions, 5) referral for patients experiencing mental disorders for follow-up and support services, and 6) patient and family centered-care approach. We will synthesize the evidence on recommendations for CPGs rated as moderate or high quality (AGREE II) (46) using a recommendation matrix based on the GRADE quality of evidence (high, moderate, low and very low) (50), recommendation strength (low, high) (47-49), health and social determinants (i.e., care equity), and whether or not recommendations were formulated using a population-based approach.(45) Matrix data will be extracted independently by pairs or reviewers for each recommendation. We will group the same or very similar recommendations published by more than one CPG. For CPGs that used a rating system other than GRADE, we will match reported categories to GRADE categories.

For selection, extraction, quality assessment and meta-synthesis, all reviewers will systematically and iteratively pilot test the process on a random selection of articles (200 citations for selection and 5 CPGs for extraction and quality evaluation and meta-synthesis in each iteration) until acceptable agreement is achieved (kappa>0.8).(32) Any discrepancies during the review process will be resolved by first consulting a senior member of the research team (WP), and then by reaching a consensus among the members of the advisory committee, if necessary.

Limitation of study

For feasibility reasons, our search strategy was not developed to systematically identify CPGs that do not specifically target mental disorders in trauma populations. Therefore, we may miss recommendations on mental health if they are included in CPGs that target trauma populations in general if no keywords relating to mental health and injury are present in the title or abstract. However, these recommendations are likely to be identified by consulting CPGs repositories and trauma association websites (Table 2), which were recognized as the best sources to find relevant guidelines.(51)

For peer review only - http://bmjopen.bmj.com/site/about/guidelines.xhtml

Potential impact

Recent data on mental health issues in trauma populations are now prompting recognized authorities (23-26) to prioritize this area to further improve the outcomes in injured patients. However, to date, there has been no synthesis of recommendations for improving mental health practices in the trauma field. This systematic review of CPGs will allow to identify recommendations with the greatest support for implementation, thus taking into account the staff shortage and time constraints omnipresent in healthcare systems. Results may be used by healthcare professionals, trauma program leaders, hospital administrators and policymakers to inform local quality improvement initiatives, identify system-wide problems and plan future actions to improve mental health practices.

Ethics and dissemination

Research ethics approval is not required, as we will conduct secondary analysis of published data. The results of our study will be disseminated in a peer-reviewed journal, at international and national scientific meetings, and an accessible summary will be distributed to stakeholders through professional, healthcare quality and persons with lived experience associations.

Author's contributions:

MB and WP led the development of the protocol and drafted the manuscript. They also secured funding and acts as guarantor for the review.

NB, MO, HTS drafted parts of the research methods, validated the search strategy, and critically appraised and approved the final manuscript.

NG, HMV assisted with the formulation of the background, objectives and inclusion criteria, and critically appraised and approved the final manuscript.

VT, MP, MM, LA, JH, HP, CG, AL, AK contributed to the development of research objectives and concept definitions, validated the search strategy, critically revised and approved the final version of the manuscript.

For peer review only - http://bmjopen.bmj.com/site/about/guidelines.xhtml

MAG and LB contributed to the formulation of research objectives and keywords, developed and tested the search strategy, and revised and approved the final version of the manuscript.

MO, MP, LA, HP, CG, WP acted as an expert in mental health and mental disorders and provided advice on how to integrate the concepts in the protocol.

Twitter:

@BrubMlanie1, @BradleyNori, @Turcottetrauma, @Alexe_Lapierre

Funding:

This work is supported by the Canadian Institutes for Health Research (#505680), Fonds de Recherche en Santé du Québec and the Strategy for Patient-Oriented Research-Quebec and the Quebec Nursing Intervention Network. Funders had not role in developing the protocol.

Competing interests:

None to declare

Patient and public involvement:

Patients with lived experience and public associations that play a role in setting standards for trauma and mental health care and in supporting persons with lived experience were involved in the design of this protocol and will be involved in the conduct, reporting, and dissemination of results associated with the proposed systematic review.

Provenance and peer reviewed:

Not commissioned; externally peer reviewed.

For peer review only

References

1. Moore L, Evans D, Hameed SM, *et al*. Mortality in Canadian Trauma Systems: A Multicenter Cohort Study. Ann Surg 2017; **265**(1): 212-17. doi: 10.1097/sla.0000000000001614
2. Truchon C, Moore L, Belcaid A, *et al*. Shaping Quality Through Vision, Structure, and Monitoring of Performance and Quality Indicators: Impact Story From the Quebec Trauma Network. Int J Technol Assess Health Care 2017;33(4):415-9. doi: 10.1017/s0266462317000198
3. World Health Organization. WHO reveals leading causes of death and disability worldwide: 2000-2019, 2020. Available: https://www.who.int/news/item/09-12-2020-who-reveals-leading-causes-of-death-and-disability-worldwide-2000-2019
4. GBD 2019 Diseases and Injuries Collaborators. Global burden of 369 diseases and injuries in 204 countries and territories, 1990–2019: a systematic analysis for the Global Burden of Disease Study 2019. Lancet 2020;396:1204-22. doi: 10.1016/S0140-6736(20)30925-9
5. Parachute. Cost of Injury in Canada, 2022. Available: https://parachute.ca/en/professional-resource/cost-of-injury-in-canada/
6. Centers for Disease Control and Prevention. Injury Prevention & Control: Cost of Injury Data, 2021. Available: https://www.cdc.gov/injury/wisqars/cost/
7. Clous EA, Beerthuizen KC, Ponsen KJ, *et al*. Trauma and psychiatric disorders: A systematic review. J Trauma Acute Care Surg 2017;82(4):794-801. doi: 10.1097/TA.0000000000001371.
8. Weinberg DS, Narayanan AS, Boden KA, *et al*. Psychiatric Illness Is Common Among Patients with Orthopaedic Polytrauma and Is Linked with Poor Outcomes. J Bone Joint Surg Am 2016;98(5):341-8. doi: 10.2106/JBJS.15.00751
9. Kruithof N, Polinder S, de Munter L, *et al*. Health status and psychological outcomes after trauma: A prospective multicenter cohort study. PLoS One 2020;15(4):e0231649. doi: 10.1371/journal.pone.0231649. eCollection 2020.
10. Wiseman T, Foster K, Curtis K. Mental health following traumatic physical injury: an integrative literature review. Injury 2013; 44(11):1383-90. doi: 10.1016/j.injury.2012.02.015. Epub 2012 Mar 10.
11. Lin W, Gong L, Xia M, Dai W. Prevalence of posttraumatic stress disorder among road traffic accident survivors: A PRISMA-compliant meta-analysis. Medicine (Baltimore) 2018;97(3):e9693. doi: 10.1097/MD.0000000000009693.
12. Roden-Foreman K, Solis J, Jones A, *et al*. Prospective Evaluation of Posttraumatic Stress Disorder and Depression in Orthopaedic Injury Patients With and Without Concomitant Traumatic Brain Injury. J Orthop Trauma 2017;**31**(9):e275-e80. doi: 10.1097/BOT.0000000000000884.
13. Joseph NM, Benedick A, Flanagan CD, *et al*. Risk Factors for Posttraumatic Stress Disorder in Acute Trauma Patients. J Orthop Trauma 2021;35(6):e209-e15. doi: 10.1097/BOT.0000000000001990.
14. van der Vlegel M, Polinder S, Toet H, *et al*. Anxiety, depression and post-traumatic stress symptoms among injury patients and the association with outcome after injury. Eur J Psychotraumatol 2022;13(1):2023422. doi: 10.1080/20008198.2021.2023422. eCollection 2022.
15. Warren AM, Jones AL, Bennett M, *et al*. Prospective Evaluation of Posttraumatic Stress Disorder in Injured Patients With and Without Orthopaedic Injury. J Orthop Trauma 2016;30(9):e305-11. doi: 10.1097/BOT.0000000000000623.
16. Bryant RA, O'Donnell ML, Creamer M, *et al*. The psychiatric sequelae of traumatic injury. Am J Psychiatry 2010;167(3):312-20. doi: 10.1176/appi.ajp.2009.09050617.

For peer review only

17. Zatzick DF, Rivara FP, Nathens, AB, *et al*. A nationwide study of post-traumatic stress after hospitalization for physical injury 2007;37(10):1469-80. https://doi.org/10.1017/S0033291707000943
18. Herrera-Escobar JP, Al Rafai SS, Seshadri AJ, *et al*. A multicenter study of post-traumatic stress disorder after injury: Mechanism matters more than injury severity. Surgery 2018;164(6):1246-50. doi: 10.1016/j.surg.2018.07.017. Epub 2018 Aug 29.
19. Evans CCD, DeWit Y, Seitz D, *et al*. Mental health outcomes after major trauma in Ontario: a population-based analysis. CMAJ 2018;190(45):E1319-e27. doi: 10.1503/cmaj.180368.
20 Mollayeva T, D'Souza A, Mollayeva S. Systematic Review of Sex and Gender Effects in Traumatic Brain Injury: Equity in Clinical and Functional Outcomes. Front Neurol 2021;12:678971. doi: 10.3389/fneur.2021.678971. eCollection 2021
21. Sharwood LN, Wiseman T, Tseris E, *et al*. Pre-existing mental disorder, clinical profile, inpatient services and costs in people hospitalised following traumatic spinal injury: a whole population record linkage study. Inj Prev 2020. doi: 10.1136/injuryprev-2019-043567
22. Herrera-Escobar JP, Seshadri AJ, Stanek E, *et al*. Mental Health Burden After Injury: It's About More than Just Posttraumatic Stress Disorder. Ann Surg 2021;274(6):e1162-e9. doi: 10.1097/SLA.0000000000003780
23. Committee on Trauma-American College of Surgeons. Best Practices Guidelines: Screening and Interventions for Mental Health Disorders and Subctance Use in the Acute Trauma Patients, 2022. Available: https://www.facs.org/quality-programs/trauma/quality/best-practices-guidelines/
24. Substance Abuse and Mental Health Services Administration. Substance Use Disorder Treatment for People With Physical and Cognitive Disabilities Advisory, 2019. Available: https://store.samhsa.gov/sites/default/files/d7/priv/pep19-02-00002_508_022620.pdf
25. Bombardier CH, Azuero CB, Fann JR, *et al*. Management of Mental Health Disorders, Substance Use Disorders, and Suicide in Adults with Spinal Cord Injury: Clinical Practice Guideline for Healthcare Providers. Top Spinal Cord Inj Rehabil 2021;27(2):152-224. doi: 10.46292/sci2702-152
26. Major Extremity trauma and Rehabilitation Consortium and the American Academy of Orthopaedic Surgeons. Evaluation of Psychosocial Factors Influencing Recovery from Adult Orthopeadic Trauma, 2019. Available: https://www.aaos.org/globalassets/quality-and-practice-resources/dod/prf-cpg-final-draft-12-3-19.pdf
27. World Health Organization. WHO global strategy on people-centred and integrated health services, 2015. Available: https://interprofessional.global/wp-content/uploads/2019/11/WHO-2015-Global-strategy-on-integrated-people-centred-health-services-2016-2026.pdf
28. Lamontagne ME, Gargaro J, Marier-Deschênes P, *et al.* A Survey of Perceived Implementation Gaps for a Clinical Practice Guideline for the Rehabilitation of Adults With Moderate to Severe Traumatic Brain Injury. J Head Trauma Rehabil 2018;33(5):306-16. doi: 10.1097/HTR.0000000000000430
29. Bulger EM, Johnson P, Parker L, *et al*. Nationwide Survey of Trauma Center Screening and Intervention Practices for Posttraumatic Stress Disorder, Firearm Violence, Mental Health, and Substance Use Disorders. J Am Coll Surg 2022;234(3):274-87. doi: 10.1097/XCS.0000000000000064
30. Sinkler MA, Furdock RJ, Vallier HA. Treating trauma more effectively: A review of psychosocial programming. Injury. 2022;53(6):1756-64. doi: 10.1016/j.injury.2022.04.02
31. Graham ID, Logan J, Harrison MB, *et al*. Lost in knowledge translation: time for a map? J Contin Educ Health Prof 2006;26(1):13-24. doi: 10.1002/chp.47
32. Higgins J TJ, Chandler J, Cumpston M, Li T, *et al*. Cochrane Handbook for Systematic Reviews of Interventions version 6.3, 2022. Available: www.training.cochrane.org/handbook

For peer review only - http://bmjopen.bmj.com/site/about/guidelines.xhtml

33. Johnston A, Kelly SE, Hsieh SC, *et al*. Systematic reviews of clinical practice guidelines: a methodological guide. J Clin Epidemiol 2019;108:64-76. doi: 10.1016/j.jclinepi.2018.11.030. Epub 2018 Dec 5.
34. Moher D, Shamseer L, Clarke M, *et al*. Preferred reporting items for systematic review and meta-analysis protocols (PRISMA-P) 2015 statement. Systematic Reviews 2015;4(1):1. doi: 10.1186/2046-4053-4-1
35. Takakusagi Y, Oike T, Shirai K, *et al*. Validation of the Reliability of Machine Translation for a Medical Article From Japanese to English Using DeepL Translator. Cureus 2021; 13(9): e17778. doi: 10.7759/cureus.17778. eCollection 2021 Sep.
36. Institute of Medicine (US) Committee on Standards for Developing Trustworthy Clinical Practice Guidelines, *Clinical Practice Guidelines We Can Trust*, ed. R. Graham, M. Mancher, and W.D. Miller. 2011: Washington (DC): National Academies Press (US).
37. American Psychiatric Association. Diagnostic and Statistical Manual of Mental Disorders. Fifth Edition, Text-Revision (DSM-TR), 2022: Washington (DC), US.
38. Blogs WB. New World Bank country classifications by income level: 2022-2023. [cited 2023 August 20th]. Available: https://blogs.worldbank.org/opendata/new-world-bank-country-classifications-income-level-2022-2023
39. Committee on Trauma-American College of Surgeons. Resources for Optimal Care of the Injured Patient, 2022. Available: https://www.facs.org/quality-programs/trauma/quality/verification-review-and-consultation-program/standards/
40. McGowan J, Sampson M, Salzwedel DM, *et al*. PRESS Peer Review of Electronic Search Strategies: 2015 Guideline Statement. J Clin Epidemiol 2016;75:40-6. doi: 10.1016/j.jclinepi.2016.01.021
41. Metha S OS, Blackport D, Teasell R. Mental Health after a Spinal Cord Injury, 2022. Available: https://scireproject.com/evidence/mental-health/introduction/
42. Kodadek LM, Freeman JJ, Tiwary D, *et al*. Alcohol-related trauma reinjury prevention with hospital-based screening in adult populations: An Eastern Association for the Surgery of Trauma evidence-based systematic review. J Trauma Acute Care Surg 2020;88(1):106-12. doi: 10.1097/ta.0000000000002501
43. Haider AH, Weygandt PL, Bentley JM, *et al*. Disparities in trauma care and outcomes in the. United States: a. systematic review and meta-analysis. J. Trauma Acute care Surg 2013;74(5);1195-205. doi: 10.1097/TA.0b013e31828c331d
44. Englum BR, Villegas C, Bolorunduro O, *et al*. Racial, ethnic, and insurance status disparities in use of posthospitalization care after trauma. J Am Coll Surg 2011;213(6):699-708. doi: 10.1016/j.jamcollsurg.2011.08.017
45. Giummarra MJ, Lennox A, Dali G, *et al*. Early psychological interventions for posttraumatic stress, depression and anxiety after traumatic injury: A systematic review and meta-analysis. Clin Psychol Rev 2018;**62**:11-36. doi: 10.1016/j.cpr.2018.05.001.
46. Brouwers MC, Kho ME, Browman GP, *et al*. AGREE II: advancing guideline development, reporting and evaluation in health care. Cmaj 2010;182(18):E839-42. doi: 10.1503/cmaj.090449
47. Ben Abdeljelil A, Freire GC, Yanchar N, *et al*. Pediatric Moderate and Severe Traumatic Brain Injury: A Systematic Review of Clinical Practice Guideline Recommendations. J Neurotrauma 2023 (ahead of print). doi: 10.1089/neu.2023.0149
48. Freire GC, Beno S, Yanchar N, *et al*. Clinical Practice Guideline Recommendations For Pediatric Multisystem Trauma Care: A Systematic Review. Ann Surg 2023 (ahead of print). doi: 10.1097/SLA.0000000000005966
49. Yanchar N, Tardif PA, Freire G, e*t al*. Clinical Practice Guidelines Recommendations for Pediatric Solid Organ Injury Care: A Systematic Review. J Trauma Acute Care Surg 2023 (ahead of print). doi: 10.1097/TA.0000000000004015

For peer review only - http://bmjopen.bmj.com/site/about/guidelines.xhtml

50. Moberg J, Oxman AD, Rosenbaum S, *et al*. The GRADE Evidence to Decision (EtD) framework for health system and public health decisions. Health Res Policy Syst 2018;16(1):45. doi: 10.1186/s12961-018-0320-2
51. Lunny C, Salzwedel DM, Liu T, *et al*. Validation of five search filters for retrieval of clinical practice guidelines produced low precision. J Clin Epidemiol 2020;117:109-16. doi: 10.1016/j.jclinepi.2019.09.022

For peer review only

Table 1. Medline (Ovid) (August 8th, 2023)

Concepts	Search strategy keywords	Research and no of results
**Trauma/ recovery** (Controlled vocabulary)	exp "Wounds and Injuries"/ or exp Brain Hemorrhage, Traumatic/ or Brain Injuries/ or Coma, Post-Head Injury/ or Craniocerebral Trauma/ or Diffuse Axonal Injury/ or exp Fractures, Bone/ or Head Injuries, Closed/ or Head Injuries, Penetrating/ or exp Intracranial Hemorrhage, Traumatic/ or exp Skull Fractures/	#1<br>1 007 620
	Fractur* OR Injur* OR TBI OR trauma*	#2<br>1 430 550
(Free text)	1 OR 2	#3<br>1 894 323
**Mental health** (Controlled vocabulary)	exp Mental Disorders/ or exp Mental Health/ or exp Substance-Related Disorders/	#4<br>1 501 202
(Free text)	"mental health" OR "mental disorder*" OR "mental illness" OR "post-traumatic stress disorder" OR "post traumatic stress disorder" OR "PTSD" OR "Acute stress disorder*" or "acute stress reaction*" or "adjustment disorder*" or "suicidal behavior*" or "self-injur*" or "self injur*" or "self-harm" or "self harm" or "depressive disorder*" or "depression" or "mood disorder*" or "anxiety disorder*" or "trauma and stressor-related disorder*" or "trauma and stressor related disorder*" or "Mental distress" or "psychological distress" OR ((drug* OR substance OR alcohol OR marijuana OR cannabis OR narcotic* OR opiate* OR opioid* OR opium) Adj4 (abus* OR "use" OR user OR usage OR misus* OR usin* OR utilis* OR depend* OR addict* OR illegal* OR illicit* OR habit* OR withdraw* OR behavi* OR abstinence* OR abstain* OR intoxica* OR addict* OR disorder*)) OR "drug rehabilitation" OR "non-prescription drugs" OR "non-prescription drug"	#5<br>1 069 467
	4 OR 5	#6<br>2 121 781
**Guideline** (Controlled vocabulary)	exp Practice Guidelines as Topic/ or exp Practice Guideline/ or exp Guidelines as Topic/ or exp Guideline/	#7<br>208 566
(Free text)	Guide OR Guideline*	#8<br>676 237
	7 OR 8	#9<br>784 520
**Total**	3 AND 6 AND 9<br>Limit to 2008-2023	3 882

For peer review only

Table 2. Preliminary list of clinical practice guidelines repositories and trauma professional associations

1-Agency for Healthcare Research and Quality	32-Guideline Central
2-Accreditation Canada	33- Guidelines International Network
3- Australasian Association for Quality in Healthcare	34-Headway—the brain injury association
4-American Academy of Orthopedic Surgeons	35- International Association for Trauma Surgery and Intensive Care
5-American Association for the Surgery on Trauma	36-International Society of Orthopaedic and Traumatology
6- American Association of Neurological Surgeons	37-Italian Society of Orthopaedics and Traumatology
7- American Board of Orthopedic Surgery	38- National Association for Healthcare Quality
8-American College of Surgeons	39-National Guidelines Clearinghouse
9-American Orthopaedic Association	40- National Institute of Health and Care Excellence
10-American Spinal Injury Association	41- National Quality Forum
11-American Trauma Society	42-Norwegian Neurosurgical Association
12-Australian Society of Orthopeadic Surgeons	43-Ontario Neurotrauma Foundation
13- Australian and New Zealand Society	44- Orthopedic Trauma Association
14-Belgian Society for Orthopedics and Traumatology	45-Scandinavian Neurotrauma Committee
15-Brain Injury Association of America	46-Scottish Intercollegiate Guidelines Network
16-Brain Trauma Foundation	47-Société Française de Chirurgie Orthopédique et Traumatologique
17- British Orthopaedic Association	48-Society of Critical Care Medicine Trauma Network
18-British Trauma Society	49-Société Royale Belge de Chirurgie Orthopédique et de Traumatologie
19- Canada's Drug and Health Technology Agency	50-Spanish Society of Orthopaedic Surgery and Traumatology
20-Centers for Disease Control and Prevention	51- Society of Trauma Nurses
21-Congress of Neurological Surgeons	52-Spinal Cord Injury Research Evidence
22- Consortium of Spinal Cord Medicine	53-Sustance Abuse and Mental Health Services Administrations

For peer review only - http://bmjopen.bmj.com/site/about/guidelines.xhtml

23-Danish Neurosurgical Society	54-Swedish Neurosurgical Societ
24-Dutch Institute for Healthcare Improvement	55-Trauma Association of Canada
25- Eastern Association for the Surgery of Trauma	56-Trauma Audit Research Network
26-European Association of Neurosurgical Societies	57-Trauma.org
27-European Federations of National Associations of Orthopaedic and Traumatology	58-U.S. Department of Health and Human Services
28-European Society for Trauma and Emergency Surgery	59-U.S. Department of Veterans Affairs
29-Finnish Association of Neuroscience Nurses	60-Western Trauma Association
30-Finnish Neurosurgical Society	61-World Health Organization
31-Greek Society of Orthopaedic and Traumatological Surgery	

For peer review only

PRISMA-P (Preferred Reporting Items for Systematic review and Meta-Analysis Protocols) 2015 checklist: recommended items to address in a systematic review protocol*

Section and topic	Item No	Checklist item	Page
**ADMINISTRATIVE INFORMATION**			
Title:			
Identification	1a	Identify the report as a protocol of a systematic review	Page 1
Update	1b	If the protocol is for an update of a previous systematic review, identify as such	NA
Registration	2	If registered, provide the name of the registry (such as PROSPERO) and registration number	Page 2
Authors:			
Contact	3a	Provide name, institutional affiliation, e-mail address of all protocol authors; provide physical mailing address of corresponding author	Page 1
Contributions	3b	Describe contributions of protocol authors and identify the guarantor of the review	Page 9
Amendments	4	If the protocol represents an amendment of a previously completed or published protocol, identify as such and list changes; otherwise, state plan for documenting important protocol amendments	NA
Support:			
Sources	5a	Indicate sources of financial or other support for the review	Page 9
Sponsor	5b	Provide name for the review funder and/or sponsor	Page 9
Role of sponsor or funder	5c	Describe roles of funder(s), sponsor(s), and/or institution(s), if any, in developing the protocol	Page 9
**INTRODUCTION**			
Rationale	6	Describe the rationale for the review in the context of what is already known	Pages 3-4
Objectives	7	Provide an explicit statement of the question(s) the review will address with reference to participants, interventions, comparators, and outcomes (PICO)	Page 5
**METHODS**			
Eligibility criteria	8	Specify the study characteristics (such as PICO, study design, setting, time frame) and report characteristics (such as years considered, language, publication status) to be used as criteria for eligibility for the review	Pages 5-6
Information sources	9	Describe all intended information sources (such as electronic databases, contact with study authors, trial registers or other grey literature sources) with planned dates of coverage	Pages 5-6
Search strategy	10	Present draft of search strategy to be used for at least one electronic database, including planned limits, such that it could be repeated	Tables 1 and 2
Study records:			

For peer review only

Data management	11a	Describe the mechanism(s) that will be used to manage records and data throughout the review	Pages 6-7
Selection process	11b	State the process that will be used for selecting studies (such as two independent reviewers) through each phase of the review (that is, screening, eligibility and inclusion in meta-analysis)	Pages 6-7
Data collection process	11c	Describe planned method of extracting data from reports (such as piloting forms, done independently, in duplicate), any processes for obtaining and confirming data from investigators	Pages 6, 7, 8
Data items	12	List and define all variables for which data will be sought (such as PICO items, funding sources), any pre-planned data assumptions and simplifications	Pages 6-7
Outcomes and prioritization	13	List and define all outcomes for which data will be sought, including prioritization of main and additional outcomes, with rationale	Page 6
Risk of bias in individual studies	14	Describe anticipated methods for assessing risk of bias of individual studies, including whether this will be done at the outcome or study level, or both; state how this information will be used in data synthesis	Page 7
Data synthesis	15a	Describe criteria under which study data will be quantitatively synthesised	Pages 7-8
	15b	If data are appropriate for quantitative synthesis, describe planned summary measures, methods of handling data and methods of combining data from studies, including any planned exploration of consistency (such as I^2, Kendall's τ)	NA
	15c	Describe any proposed additional analyses (such as sensitivity or subgroup analyses, meta-regression)	Page 8
	15d	If quantitative synthesis is not appropriate, describe the type of summary planned	Page 8
Meta-bias(es)	16	Specify any planned assessment of meta-bias(es) (such as publication bias across studies, selective reporting within studies)	NA
Confidence in cumulative evidence	17	Describe how the strength of the body of evidence will be assessed (such as GRADE)	NA

*** It is strongly recommended that this checklist be read in conjunction with the PRISMA-P Explanation and Elaboration (cite when available) for important clarification on the items. Amendments to a review protocol should be tracked and dated.** **The copyright for PRISMA-P (including checklist) is held by the PRISMA-P Group and is distributed under a Creative Commons Attribution Licence 4.0.**

From: Shamseer L, Moher D, Clarke M, Ghersi D, Liberati A, Petticrew M, Shekelle P, Stewart L, PRISMA-P Group. Preferred reporting items for systematic review and meta-analysis protocols (PRISMA-P) 2015: elaboration and explanation. BMJ. 2015 Jan 2;349(jan02 1):g7647.

For peer review only
